# The effect of preanalytical factors on cerebrospinal fluid and plasma proteomics: a systematic experimental study

**DOI:** 10.1186/s12014-026-09604-5

**Published:** 2026-05-22

**Authors:** Megumi Tatsumi, Tomoko Miyakawa, Takako Enokida, Haruna Kaneko, Kosuke Saito, Shinsuke Hidese, Yuji Takahashi, Yuichi Goto, Hiroshi Kunugi, Kotaro Hattori

**Affiliations:** 1 Department of Biobanking, Medical Genome Center, National Center of Neurology and Psychiatry, 4-1-1 Ogawa-Higashi, Kodaira, 187-8551 Tokyo Japan; 2 Division of Medical Safety Science, National Institute of Health Sciences, 3-25-26 Tonomachi, Kawasaki-ku, Kawasaki, 210-9501 Kanagawa Japan; 3 Department of Psychiatry, Teikyo University School of Medicine, 2-11-1 Kaga, Itabashi-ku, 173-8605 Tokyo Japan; 4 Department of Neurology, National Center of Neurology and Psychiatry Hospital, 4-1- 1 Ogawa-Higashi, Kodaira, 187-8551 Tokyo Japan

**Keywords:** Pre-analytical variables (PAVs), Cerebrospinal fluid (CSF), Biobanking, Sample handling, SomaScan, Storage temperature, Freeze-thaw cycles, Quality control, Robustness

## Abstract

**Background:**

Preanalytical variations (PAVs) are a major source of irreproducibility in biomarker studies. While their effects on blood have been widely examined, their impact on cerebrospinal fluid (CSF) proteomics remains less well characterized. We systematically evaluated common PAVs to inform biobanking and biomarker research.

**Methods:**

CSF and plasma samples were collected at an ISO 20387–accredited biobank. For each condition, three or four independent paired samples were analyzed. PAVs included storage temperature, processing delay, centrifugation speed, storage tube type, freeze–thaw cycles, and blood contamination. Protein abundance was quantified using SomaScan. Selected analytes were further evaluated using orthogonal immunoassays. Fold changes were calculated as linear RFU ratios relative to baseline. Paired t-tests with false discovery rate (FDR) correction were performed. Analytes showing detectable or larger changes were defined based on predefined fold-change thresholds with nominal *p* < 0.05.

**Results:**

Plasma proteomes were highly sensitive to delays and temperature, with 969 analytes showing large changes after 24 h at 25 °C (counts refer to large changes), compared to 26 analytes in CSF. At 4 °C, CSF remained stable for 24 h (4 changes), whereas plasma showed extensive changes after 24 h (465) and 72 h (813). Storage at − 20 °C caused substantial shifts in both matrices (CSF: 144; plasma: 1,164), whereas no analytes differed in plasma stored at − 50 °C relative to − 80 °C. Freeze–thaw cycles up to three resulted in few altered analytes, whereas 11 cycles led to pronounced changes (CSF: 198; plasma: 21). Hemolysis in plasma induced 28 changes, whereas CSF blood contamination ≤ 500 cells/mm^3^ was associated with few changes. After FDR correction, no analytes remained significant in CSF, whereas multiple analytes remained significant in plasma under selected conditions.

**Conclusions:**

Storage temperature and processing delay were the most influential PAVs, with plasma more susceptible than CSF. For optimal quality, CSF should be processed within 4 h or kept at 4 °C, plasma within 2 h, and both stored at ≤ − 80 °C. Freeze–thaw cycles should be limited to three.

**Supplementary Information:**

The online version contains supplementary material available at 10.1186/s12014-026-09604-5.

## Introduction

Cerebrospinal fluid (CSF) is a biological fluid that exists around the brain and spinal cord, with a volume of approximately 150 mL. It is continuous with brain interstitial fluid and contains brain-derived molecules. However, the blood-brain barrier prevents the free exchange of molecules between the CSF and systemic circulation. Owing to these characteristics, CSF is an excellent clinical sample for brain diseases. Amyloid-β and phosphorylated tau (p-tau) are established biomarkers for the biological definition of Alzheimer’s disease, while neurofilament light chain (NfL) reflects neuroaxonal damage and disease severity rather than disease specificity [1].

Pre-analytical variables (PAVs) significantly impact biomarker research reproducibility, accounting for the largest component of total error in clinical laboratories [2]. These variables include sample collection, handling, processing, and storage [3]. Studies on Alzheimer’s disease biomarkers have demonstrated that factors such as room temperature exposure time, blood contamination, and dietary effects have minimal impact on CSF, whereas the type of storage tube material significantly influences results [4, 5]. Furthermore, research groups on multiple sclerosis have proposed consensus guidelines based on an investigation of collection practices across institutions [6]. However, to our knowledge, the large spectrum of pre-analytical conditions for CSF proteomic analysis has not been comprehensively analyzed.

Pre-analytical variables also significantly affect blood proteomics, influencing protein stability and overall proteome quality. The collection, handling, and storage of blood samples are critical factors that can impact the results [7, 8].

In recent years, demand for the standardization of biobanks has been increasing, leading to the publication of international standards such as ISO 20387:2018, “General Requirements for Biobanking.” ISO 21899:2020, “General Requirements for the Validation and Verification of Processing Methods for Biological Material in Biobanks,” is one such related standard, issued to guide the validation and verification of sample processing conditions. This standard emphasizes the need to analyze the robustness of these conditions and their impact on the intended use of samples.

Recent advances in omics technologies, such as SomaScan proteomics, now allow for the measurement of more than 7,000 analytes with high precision [9]. To develop accurate biomarkers using this technology, incorporating PAV considerations into statistical analyses of existing samples and optimizing the processing methods for prospective sample collections based on PAVs is essential. This study aims to investigate the impact of different processing conditions, from sample collection to analysis, on CSF and blood proteomic data.

## Materials and methods

### Sample collection

Volunteers were recruited and CSF and plasma samples were collected at the NCNP Biobank, which was recently accredited with ISO 20387, following previously described methods [10]. Written informed consent was obtained from every participant. This study was conducted in accordance with the Declaration of Helsinki and was approved by the ethics committee of NCNP, Japan (A2019-092).

### Materials

The following collection tubes were used in the study: VENOJECT II, VP-NA050K (Terumo Corp., Japan) and INSEPACK II-D, 535536 (Sekisui Medical, Japan) for blood collection; a Screw cap tube 15 ml tube, 62.554.001MPCS (Sarstedt, Japan), and PROTEOSAVE™ Conical tube 15mL, MS-52150 (Sumitomo Bakelite, Japan) for CSF collection with low protein binding; and Falcon 15 mL Conical Centrifuge Tubes, 352096 (Corning, Japan) and Falcon 16 mL Round Bottom Polystyrene Test Tube, 352037 (Corning, Japan) for CSF collection with normal protein binding.

The following storage tubes (primary container) were used in the study:

96 Jacket tubes 1.3 mL tube, JRDPS-2 M (FCR & Bio Co., Ltd, Japan) and PROTEOSAVE Slimtube 1.5 mL, MS-4202X (Sumitomo Bakelite, Japan) for CSF storage; and 96 Jacket Tubes 1.0 mL, WJRDS-2 M (FCR & Bio Co., Ltd, Japan), and Matrix 500 µL ScrewTop Tubes, 3744 (Thermo Fisher Scientific) for plasma storage.

### Compliance with ISO 21899

The methodology described in this paper adheres to several key aspects of ISO 21899:2020. Outsourced testing was selected in accordance with section 8.4.3, and SomaLogic Inc. is ISO 15189 accredited. Robustness was tested in accordance with section 8.5.4.

### Evaluated factors

As outlined below, the experimental factors suspected of causing variation during everyday processing, or known/suspected to impact the outcome of processing methods, were altered to assess their impact. For each condition, experiments were conducted with *N* = 3 for CSF and *N* = 4 for plasma (*N* = 3 for hemolysis). Unless specified otherwise, the aliquot volume used was 1 mL for all cases (Table 1).

**Table 1:** Summary of the compared processing conditions

Blood contamination	CSF	Plasma
	~ 10, 100, 500, 5000/mm^3^	-
Collection tubes	**Low protein binding**, polypropylene, polystyrene	**Venoject**, Insepack
Hemolysis	-	**Negative**, positive
Time-to-centrifuge (4 and 25 °C)	4 °C: **0 h**, 2 h, 4 h, 24 h 25 °C: 2 h, 4 h, 24 h	4 °C: **0.5 h**, 2 h, 24 h, 72 h 25 °C: **0.5 h**, 2 h, 24 h
Centrifuge speed	1000 g, **2000 g**, 4000 g	-
Storage tubes	**Low protein binding**, polypropylene	**96 Jacket tubes**, matrix tube
Storage temperature	-20 °C, **-80°**C	-20 °C, -50 °C, **-80°**C
Freeze thaw cycles	**1**, 2, 3, 11	**1**, 2, 3, 11

The bold and underlined conditions are the default conditions and serve the baseline for comparison with other conditions

### Replicate structure

All experiments were conducted using biological replicates, in which samples obtained from the same donor were subjected to different pre-analytical conditions (e.g., blood contamination levels, storage temperature and duration, and freeze–thaw cycles), enabling paired comparisons across conditions. The number of biological replicates per condition was *N* = 3 for CSF and *N* = 4 for plasma (*N* = 3 for hemolysis), as described above. Each condition was measured once per sample using the SomaScan assay; thus, observed variability reflects a combination of biological variation and assay-related variability rather than technical replication within the assay. Analytical reproducibility was evaluated using pooled samples. Five aliquots prepared from the same pooled samples were independently processed and measured by SomaLogic, and coefficients of variation (CVs) were calculated for each analyte.

### CSF

#### Blood contamination

Blood samples (EDTA2Na) were collected from volunteers, and the red blood cell count was immediately measured. The blood was diluted with saline and then added to CSF to achieve final concentrations of 100, 500, or 5000 cells/mm^3^. The red blood cell count was measured again to ensure that the concentration was within ± 20% of the target value. The mixture was then allowed to stand for 10 min before further processing.

#### Type of collection tubes

CSF collected in low protein-binding tubes was aliquoted into low protein-binding tubes, polypropylene tubes, and polystyrene tubes, then left to stand at 25 °C for 2 h before processing.

#### Time and temperature from collection to processing

After collection, CSF samples were left to stand at either 4–25 °C for 0, 2, 4, or 24 h, followed by processing.

#### Centrifugation conditions

The samples were centrifuged at 1000 g, 2000 g, and 4000 g for 10 min each, and the supernatant was stored.

#### Long-term storage tube types

CSF samples processed under the same conditions were aliquoted into polypropylene tubes (96 Jacket tubes, FCR & Bio) and low protein-binding tubes (PROTEOSAVE SS 1.5 mL Slimtube, Sumitomo Bakelite), with 110 µL per tube, and stored between December 2016 and December 2021.

#### Storage temperature

CSF aliquoted at 110 µL into low protein-binding tubes was initially stored at either − 80 °C or -20 °C for 4 weeks, then transferred to and stored at -80 °C.

Freeze-thaw cycles: Four low protein-binding tubes containing 110 µL of frozen CSF were subjected to 0, 1, 2, or 10 additional freeze-thaw cycles prior to measurement.

### Plasma

#### Blood collection tube types

The widely used blood collection tubes in Japanese biobanks (VENOJECT II, Terumo Corp.) and the newer safety cap blood collection tubes (Insepack, Sekisui Medical) were used for blood collection from the same volunteer and 5 mL of blood was collected in each tube.

#### Hemolysis

Hemolysis can occur because of insufficient blood collection in vacuum tubes or when using syringes. To assess the impact of hemolysis on the proteome, artificial hemolysis was induced by vortexing for approximately 2 min and compared with non-hemolyzed samples. Hemolysis was visually confirmed by a clinical laboratory technician at the biobank.

#### Time and temperature from collection to processing

After plasma collection, samples were left at 4–25 °C for 0.5, 2, 24 h, and at 4 °C for 72 h before centrifugation.

#### Storage tube types

After centrifugation, plasma was aliquoted into 96 Jacket tubes (FCR & Bio) and Matrix tubes (Thermo Fisher Japan), with 130 µL per tube, and stored.

Storage temperature: Plasma aliquoted at 130 µL was stored at -80 °C, -50 °C, or -20 °C for 4 weeks after aliquoting, and subsequently transported at -80 °C for measurements.

#### Freeze-thaw cycles

Aliquoted plasma with a volume of 130 µL was subjected to 1, 2, 3, and 11 freeze-thaw cycles, followed by measurements.

### SOMAscan proteome analyses

Proteome analysis was conducted by SomaLogic Inc. (USA) using the SomaScan^®^ 7k assay (version v4.1). This platform is described as targeting 7,288 human proteins [9]. All analyses were performed using the analyte-level data as provided. SomaScan data were normalized using the standard SomaLogic normalization pipeline, including hybridization control normalization, median signal normalization, and calibration (plate scaling), as previously described [11]. The resulting relative fluorescence unit (RFU) values were used for downstream analyses.

Detailed analyte-level performance metrics for the SomaScan assay (including coefficients of variation, signal-to-noise characteristics, and LoD estimates) are available through the SomaScan menu portal (SomaLogic Inc.) upon registration.

To ensure analytical robustness within our dataset, we performed repeated measurements of pooled CSF and plasma samples (*n* = 5 technical replicates) and calculated coefficients of variation (CVs) for each analyte. Only analytes with CV < 15% were included in downstream analyses.

### ELISA

For orthogonal validation, ELISA assays were performed for fibrinogen and IL-8 using commercially available kits according to the manufacturers’ instructions. Fibrinogen levels were measured using a Human Fibrinogen ELISA kit (Abcam, ab108841). CSF samples were diluted 1:50 prior to analysis. IL-8 levels were measured using a Human IL-8/CXCL8 DuoSet ELISA (R&D Systems, DY208). Samples were analyzed without dilution. These analytes were selected based on (i) robust changes observed in the SomaScan analysis and (ii) suitability of the assay sensitivity for the expected concentration range. Total protein levels were measured using the Pierce BCA Protein Assay Kit (Thermo Fisher Scientific, 23225) according to the manufacturer’s instructions.

### Statistical analysis

Analytical reproducibility was evaluated using precision metrics. Five aliquots prepared from the same pooled samples were independently processed and measured by SomaLogic, and coefficients of variation (CVs) were calculated for each analyte. To ensure analytical reliability, only analytes with a CV < 15% were included in downstream statistical analyses. Given that a CV threshold of 20% is commonly used to define the limit of quantification (LoQ), the majority of analyzed analytes are considered to fall within the quantifiable range. Fold changes were calculated as linear RFU ratios relative to the corresponding baseline condition (RFU_test / RFU_baseline), without log_2_ transformation. Statistical comparisons were performed using paired t-tests, as samples were aliquoted from the same original specimen and subjected to different experimental conditions. This paired design increases sensitivity to detect condition-specific changes despite the limited sample size. Analytes were classified based on predefined fold-change thresholds. Moderate changes were defined as |log_2_ fold change| > 0.5 (corresponding to fold changes > 1.41 or < 0.71), and larger changes as |log_2_ fold change| > 1 (corresponding to fold changes > 2 or < 0.5), together with a nominal *p* < 0.05 (Figure S1). Given the exploratory nature of this study and the limited sample size, nominal p-values were used for primary screening to prioritize sensitivity. To provide a more conservative assessment, p-values were additionally adjusted for multiple testing using the Benjamini–Hochberg false discovery rate (FDR). For visualization, volcano plots were generated by plotting log_2_-transformed fold changes against −log_10_-transformed p-values. Given the large number of analytes and the limited sample size, the statistical analyses were intended to be exploratory and hypothesis-generating rather than definitive for individual proteins.

### Human protein atlas–based selection of blood proteins

To contextualize the effects of blood contamination, highly abundant blood proteins were selected using publicly available data from the Human Protein Atlas (HPA). Blood protein abundance data based on immunoassay measurements were obtained from the HPA blood proteome dataset. Proteins measurable on the SomaScan platform were identified by matching gene symbols between the HPA dataset and the SomaScan target list. These proteins were ranked according to blood abundance, and the top 10 were selected for further analysis. For reference, RNA expression data across multiple brain regions were obtained from the HPA tissue expression database. Region-specific brain expression values were summarized as the median across brain regions. Brain RNA expression data were included for contextual interpretation only and were not used as selection criteria. The selected proteins were evaluated under controlled blood contamination conditions (100, 500, and 5,000 red blood cells/mm^3^), as summarized in Supplementary Table S1.

## Results

### Assay precision

Pooled samples were measured 5 times, yielding median CVs of 4.8% and 3.5% for CSF and plasma, respectively. Measurements with a CV less than 15% were used for statistical analyses, which correspond to 7001 for CSF and 7044 for plasma.

### CSF: blood contamination

Blood contamination can occur during CSF collection. Therefore, we analyzed the effect of blood contamination on the CSF proteome. When autologous blood was added to the CSF, no analytes met the predefined criteria for change (fold changes |log2 fold change| > 0.5 and a nominal *p* < 0.05) at a blood contamination level of 100 cells/mm³ compared with CSF without blood contamination.

At a blood contamination level of 500 cells/mm³, 2 analytes met the predefined criteria for change, and none met the criteria for large changes (fold changes |log2 fold change| > 1 and a nominal *p* < 0.05) (Figure S2, Table S2).

At 5,000 cells/mm³, visible blood contamination was confirmed, and 34 analytes met the criteria for change, including 11 that met the criteria for large changes. We observed limited overlap between contamination levels. Of the two analytes identified at 500 cells/mm³, one (C4b-binding protein alpha chain) was also significantly increased at 5,000 cells/mm³, while the other (hemoglobin) showed a marked increase but did not reach statistical significance at the higher contamination level due to increased variability. Among these, the antimicrobial protein LL-37 exhibited the largest relative change among the affected analytes under this controlled blood-spiking condition (Figure S3A). While independent validation of LL-37 would be desirable, this was not feasible due to sensitivity limitations of currently available assays for CSF measurements. To provide independent validation of SomaScan measurements, we performed orthogonal ELISA assays. Fibrinogen levels showed strong concordance between SomaScan and ELISA (*R* = 0.88). Consistent with the SomaScan results, fibrinogen concentrations increased with blood contamination. Although the magnitude of change differed between platforms, the direction of change was consistent (Figure S3B and S3C). To characterize the response of blood-derived proteins to contamination, we examined highly abundant blood proteins using data from the Human Protein Atlas. Most major blood proteins showed only modest fold changes even at the highest contamination level (Table S1). The distributions of coefficients of variation (CVs) were broadly comparable across different levels of blood contamination (Figure S4). No statistically significant differences were observed across conditions (Kruskal–Wallis test, *p* = 0.29). Total protein levels increased by an average of 4.7% at a contamination level of 5,000 cells/mm³.

### CSF: collection tubes

CSF was collected in polystyrene, polypropylene, and low protein-binding treated tubes. Compared with low protein-binding tubes, CSF collected in polypropylene tubes showed 2 analytes showed moderate changes (Figure S5).

### Plasma: plasma collection tubes

Because different facilities use different blood collection tubes, we compared two commonly used blood collection tubes for plasma in Japan. Under this condition, no analytes showed measurable changes (Figure S5).

### Plasma: effect of hemolysis

To identify the analytes affected by hemolysis, artificial hemolysis was induced using a vortex mixer and compared with the original samples. Under this condition, 54 analytes showed measurable changes, including 28 analytes showing large changes (Figure S6, Table S3). Although hemoglobin was increased by 8.2 times, it was excluded from the analysis owing to a high CV of 17.8%. Some analytes were also decreased; for example, haptoglobin was found to decrease by half.

### CSF: time and temperature from collection to processing

**Fig. 1:**
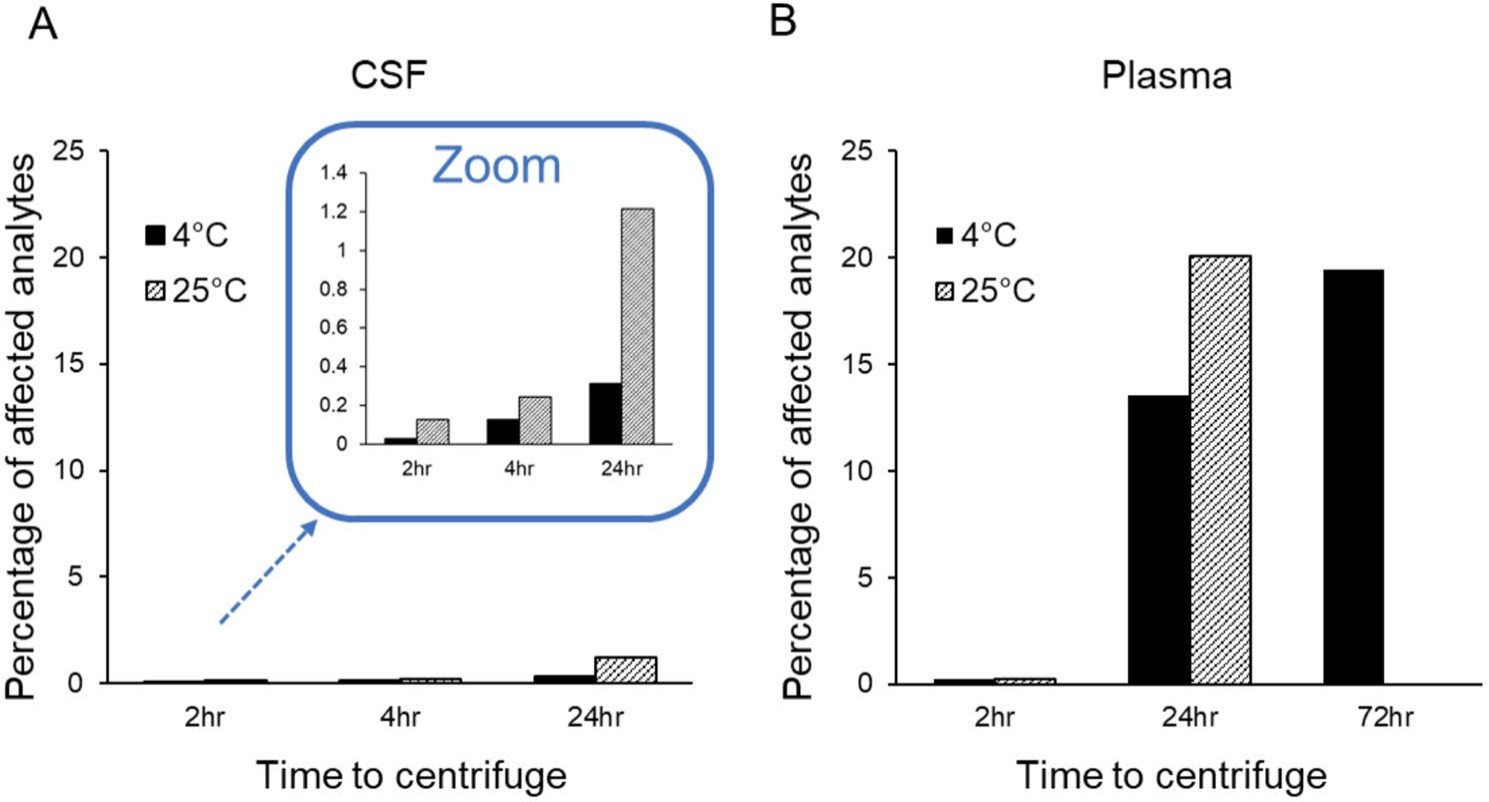
Effect of time and temperature before processing. (**A**) CSF samples were stored at either 4–25 °C and processed immediately (< 15 min) or after 2, 4, and 24 h. The y-axis represents the percentage of analytes that met the predefined criteria for change (fold-change thresholds with a nominal *p* < 0.05) relative to the immediately processed sample under each condition. Due to the smaller magnitude of changes in CSF, an inset with an expanded y-axis scale is provided to improve visualization. (**B**) After plasma collection, samples were stored at 4–25 °C for 0.5, 2, or 24 h, and at 4 °C for 72 h before centrifugation. The y-axis represents the percentage of analytes that showed measurable changes relative to the immediately processed sample under each condition

The effects of different storage times before processing at 4 °C and 25 °C on the CSF proteome were examined. At 4 °C, 2 analytes (0 large change) changed after 2 h, 9 (4 large changes) after 4 h, and 22 (4 large changes) after 24 h. At 25 °C, the corresponding numbers were 9 (3 large changes) after 2 h, 17 (8 large changes) after 4 h, and 85 (26 large changes) after 24 h (Fig. 1A, Figure S7). Of the 85 analytes that changed at 25 °C for 24 h, 57 (18 large changes) decreased, and 28 (8 large changes) increased (Table S4).

### Plasma: time and temperature from collection to processing

Plasma proteome changes due to different temporal storage conditions were observed. At 4 °C, 16 (7 large changes) analytes changed after 2 h, 953 (465 large changes) after 24 h, and 1,367 (813 large changes) after 72 h. At 25 °C, 17 (2 large changes) analytes changed after 2 h, and 1,415 (969 large changes) after 24 h (Fig. 1B, Figure S8). Of the 1,415 analytes that changed at 25 °C for 24 h, 95 (31 large changes) decreased, and 1,322 (939 large changes) increased (Table S5). After FDR correction, 157 analytes remained significant (q < 0.05) under the 25 °C 24-hour condition, providing a more conservative assessment.

GO enrichment analysis of proteins altered after 24 h incubation at room temperature revealed significant enrichment in processes related to translation, RNA metabolism, vesicle-mediated transport, endosomal organization, and protein catabolic pathways (Figure S9, Table S6). In addition, pathways associated with extracellular vesicle biogenesis and platelet-related processes were also enriched. To provide independent validation of SomaScan measurements, we performed orthogonal ELISA assays. IL-8 levels under different pre-centrifugation delay and temperature conditions showed moderate concordance between SomaScan and ELISA (*R* = 0.65). Consistent with the SomaScan results, IL-8 concentrations increased with prolonged pre-centrifugation delay. Although the magnitude of change differed between platforms, the direction of change was consistent (Figure S10).

### CSF: centrifugation conditions

No analytes met the predefined criteria for change when CSF samples centrifuged at 1000–4000 g for 10 min were compared with the default condition (2000 g) (Figure S11).

### CSF: long-term storage tubes

Low protein-binding treated tubes and untreated polypropylene tubes were compared after 5 years of storage at -80 °C. Eight analytes showed measurable changes in the untreated tubes compared with the low protein-binding tubes, five of which showed decreased levels (Figure S12). No analytes met the criteria for a large change.

### Plasma: storage tubes

Among two plasma storage tube brands, one analyte showed moderate change: Tumor protein p53-inducible protein 11 (SeqID: 13022-20; SomaID: SL019515; UniProt ID: O14683), with a fold change of 1.4 (*p* = 0.02) (Figure S12). No analytes met the criteria for a large change.

### CSF: storage temperature

**Fig. 2:**
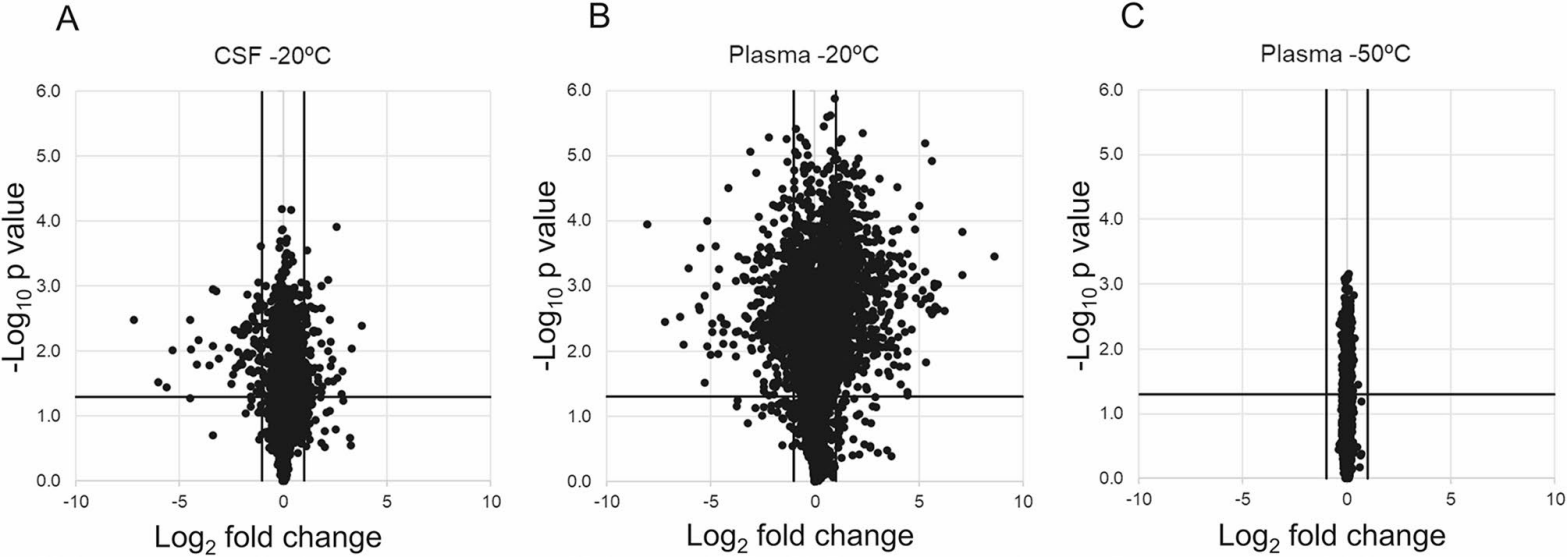
Impact of storage temperature on CSF and blood proteomes analyzed by volcano plots. (**A**) Comparison of the CSF proteome between samples stored at -80 °C and − 20 °C for 4 weeks using volcano plot. The y-axis represents the negative logarithm of the p-value from the t-test, while the x-axis indicates the logarithm of the fold change between two conditions. (**B**) Volcano plot comparing the blood proteome of samples stored at -20 °C versus − 80 °C. (**C**) Volcano plot comparing the blood proteome of samples stored at -50 °C versus − 80 °C

A comparison of CSF storage at -80 °C and − 20 °C for 4 weeks revealed that 349 analytes showed measurable changes at − 20 °C relative to − 80 °C, of which 144 also met the criteria for large changes (Fig. 2A, Table S7). Among these, 234 analytes (70 with large changes) increased and 115 analytes (74 with large changes) decreased at − 20 °C. Notably, some analytes, such as Transmembrane Protein 132 A (SeqID: 7871-16), exhibited a pronounced decrease (> 99%) compared with samples stored at − 80 °C.

To further assess whether specific biological processes were preferentially affected, we performed Gene Ontology enrichment analysis on proteins showing large changes. However, no GO terms reached statistical significance after multiple testing correction in either CSF or plasma.

### Plasma: storage temperature

A comparison of plasma storage temperatures (-80 °C, -50 °C, -20 °C) for 4 weeks showed that at -50 °C, only one molecule showed measurable change relative to -80 °C (SeqID: 2683-1, SomaID: SL000456, Protein name: Complement C3b, inactivated, UniprotID: P01024, fold change: 1.46, *p* = 0.036, Fig. 2C). In contrast, storage at -20 °C resulted in 2,217 analytes meeting the criteria for change, including 1,164 that also met the criteria for large changes, with 1,268 analytes (774 large changes) increasing and 949 analytes (390 large changes) decreasing (Fig. 2B, Table S8). After FDR correction, 2,124 analytes remained significant (q < 0.05) under the − 20 °C condition, providing a more conservative assessment. Some analytes, such as Chromogranin-A, showed no detectable change at -50 °C but decreased by less than 1.5% at -20 °C.

### CSF: freeze-thaw cycles

**Fig. 3:**
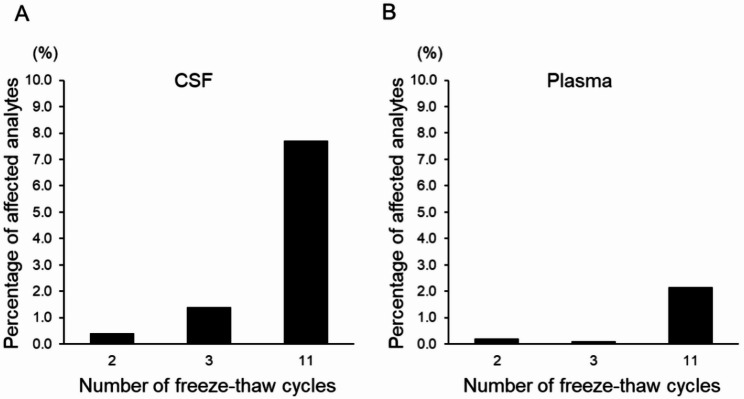
Effect of repeated freeze-thaw cycles on CSF and plasma proteomes. (**A**) CSF samples. The y-axis represents the percentage of analytes that showed measurable changes, including both increases and decreases, compared with samples subjected to a single freeze–thaw cycle. (**B**) Plasma samples analyzed under the same freeze–thaw conditions and criteria as in (**A**)

The impact of freeze-thaw cycles was examined by comparing samples subjected to multiple cycles with those subjected to a single freeze–thaw cycle (thawed immediately before analysis). Under predefined criteria, 29 analytes (including 1 with a large change) showed measurable changes after two cycles, 99 analytes (including 10 with large changes) after three cycles, and 563 analytes (including 198 with large changes) after 11 cycles (Fig. 3A, Figure S13, Table S9).

### Plasma: freeze-thaw cycles

The impact of freeze-thaw cycles on plasma was investigated. Compared with samples subjected to a single freeze–thaw cycle, 14 analytes showed measurable changes after two cycles, 8 analytes after three cycles, and 152 analytes after 11 cycles (Fig. 3B). Among these, no analytes met the criteria for a large change after two cycles, whereas 1 analyte after three cycles and 21 analytes after 11 cycles met the criteria for a large change (Fig. 3B, Figure S13, Table S10). To assess whether freeze–thaw–induced changes are randomly distributed or biologically structured, we performed Gene Ontology (GO) enrichment analyses for significantly altered proteins in both CSF and plasma (Figure S14-S15, Table S11-S13). In CSF, enriched GO terms were primarily related to glycoprotein biosynthesis, glycosylation, and carbohydrate metabolic processes, suggesting that proteins involved in post-translational modification may be particularly sensitive to freeze–thaw stress. In plasma, a limited number of GO terms were enriched, including categories annotated as axonal or vesicle transport. These terms were driven by a small number of proteins (e.g., BORCS5, BLOC1S1, and ARL8A), which are components of general vesicle and lysosomal transport machinery rather than neuron-specific processes. To further explore the underlying mechanisms, we performed an additional enrichment analysis restricted to proteins showing increased abundance after freeze–thaw cycles (Figure S15). This analysis revealed enrichment of processes related to vesicle organization and intracellular transport.

## Discussion

This study comprehensively examined the effects of PAV on CSF and plasma proteomes, focusing on their impact on the stability and reliability of proteome data. The results revealed significant effects of factors such as time and temperature before processing, storage temperature, freeze-thaw cycles, and hemolysis on the proteome profiles of the samples.

### Impact of blood contamination on CSF proteome

Blood contamination in CSF has been reported to impact the analysis of various analytes, as even minor contamination can alter the protein profile and potentially confound biomarker identification [12]. Hemoglobin and other blood-derived proteins have been widely used as indicators of blood contamination in CSF analyses, allowing for the assessment of blood intrusion in CSF samples [13, 14]. The degree of contamination affects different molecular classes variably; for instance, certain biogenic amines and vitamins show reduced concentrations upon red blood cell (RBC) lysis, while others show increased levels because of intracellular release [14, 15]. Further, previous studies indicate that CSF-targeted metabolomic and proteomic analyses remain feasible when RBCs are effectively removed by centrifugation prior to freezing [14]. In the literature, an erythrocyte count above approximately 500 cells/mm^3^ is generally considered a threshold above which CSF samples may not be suitable for biomarker analyses [16]. Higher levels (e.g., ≥ 5,000 cells/mm^3^) have been reported to affect certain protein measurements, such as immunoglobulins [17]. In this study, when RBC contamination remained below 500 cells/mm^3^, few analytes met the predefined statistical and fold-change criteria for being classified as altered under the current sample size and exploratory analysis thresholds. However, at contamination levels exceeding 5000 cells/mm^3^, a notable increase in certain proteins was detected. These findings suggest that although minor contamination does not significantly interfere with CSF proteome analysis, substantial RBC contamination may introduce artifacts that could compromise biomarker discovery. The selective impact of blood contamination on the CSF proteome can be explained, at least in part, by differences in baseline protein concentrations between blood and CSF. In general, protein concentrations in blood are approximately 100–200-fold higher than in CSF. In the present study, a contamination level of 5,000 cells/mm³ corresponds to only a small fraction of circulating blood cell counts, and thus is unlikely to substantially alter the abundance of most proteins. However, proteins with particularly large concentration gradients between blood and CSF are more susceptible to contamination effects. For example, fibrinogen is present at approximately 1,000–2,000 µg/mL in blood but at around 0.5 µg/mL in CSF [10], representing a difference of several thousand-fold. This large gradient likely explains its consistent increase with blood contamination. Similarly, LL-37 is present in blood at measurable concentrations but is typically undetectable in normal CSF [18], suggesting a substantial concentration difference that may underlie its apparent sensitivity to contamination. These observations indicate that the impact of blood contamination is primarily driven by relative concentration differences between compartments, rather than by protein class per se.

### CSF: type of collection tubes

The choice of collection tubes is reported to impact the measurement of CSF biomarkers, particularly amyloid-β peptides and tau proteins, which are critical for diagnosing Alzheimer’s disease [19, 20]. Previous studies have reported that non-specific binding of proteins to tube surfaces can substantially alter CSF protein profiles, thereby affecting biomarker quantification 12, 20]. However, in our analysis, few analytes met the predefined statistical and fold-change criteria when comparing CSF samples collected using different tube materials. Notably, the aforementioned studies assessed the effects of both collection and extended storage conditions. This suggests that although middle or long-term storage in different container materials may influence biomarker stability, temporal short-term storage during the collection phase (~ 2 h) does not appear to cause major alterations under the short-term collection conditions examined in this study.

### Plasma: plasma collection tubes

Research indicates that differences between manufacturers of blood collection tubes can influence certain hematological test results, although the clinical significance of these variations is often minimal. Previous studies have reported statistically significant differences in parameters such as hematocrit, mean corpuscular volume, and platelet distribution width when comparing tubes from different brands [21, 22]. However, these variations generally remain within acceptable total error limits and do not affect clinical decision-making [23, 24]. Similarly, in our study, no significant differences were detected in proteomic analysis results when comparing plasma collection tubes from different manufacturers.

### Plasma: effect of hemolysis

Hemolysis significantly impacts the proteome and biomarker discovery in blood samples by introducing extraneous proteins, particularly hemoglobin, which can interfere with biomarker detection and validation [25]. In our analysis, we observed a marked increase in hemoglobin and other erythrocyte-derived analytes following hemolysis. We also found a notable decrease in haptoglobin levels, likely because of its secondary consumption in response to increased hemoglobin levels [26].

### CSF: time and temperature from collection to processing

Pre-centrifugation handling of CSF affects its molecular composition and stability. Delays before processing can alter protein levels and metabolite concentrations, potentially confounding biomarker analysis [27, 28]. Time and temperature are particularly critical, influencing the stability of key CSF proteins such as tau, phosphorylated tau (P-tau), and transthyretin [29]. Additionally, processing delays can significantly affect inflammation-related protein biomarkers [28].

We evaluated the impact of short-term holding temperature (4 °C vs. 25 °C) and pre-processing time on the CSF proteome. Under the predefined statistical and fold-change criteria, the number of analytes showing changes in CSF increased with longer holding times, particularly at 25 °C, while remaining limited across conditions under the predefined statistical and fold-change criteria. These results suggest that, within the scope of this study, CSF proteomes exhibited relatively limited changes, particularly when samples were held at 4 °C. When immediate processing is not feasible, temporary storage at 4 °C may help reduce pre-analytical variability and support the reliability of downstream CSF proteomic measurements.

### Plasma: time and temperature from collection to processing

Processing delays and elevated temperatures induce extensive changes, particularly in the plasma proteome. Leaving whole blood at room temperature for 24 h has been reported to cause a 2- to 10-fold increase in the levels of inflammation-related proteins such as caspase 8, IL-8, IL-18, and SIRT2 [28]. Our study confirmed a significant rise in IL-8, which was corroborated by orthogonal validation via ELISA. Additionally, studies have shown that centrifugation at cold temperatures (e.g., 8 °C) can lead to higher cytokine levels than processing at room temperature, a phenomenon attributed to cold-induced platelet activation and the subsequent release of stored compounds [30]. Increased levels of cytoskeletal proteins such as actin beta (ACTB) and vinculin (VCL) serve as robust markers of the intracellular leakage associated with these processing delays [31]. Consistent with this mechanism, both our data and previous literature have identified significant increases in S100A family proteins, including S100A8 and S100A9, as a function of processing delay at RT [31]. Delayed processing of whole blood at room temperature resulted in coordinated changes in proteins, with GO enrichment in pathways related to translation, RNA metabolism, vesicle-mediated transport, and protein catabolic processes. These patterns are not consistent with passive degradation alone, but instead indicate substantial contributions from blood cells present in the sample. Because the samples were maintained as whole blood without centrifugation, blood cells (including platelets and leukocytes) likely remained metabolically active during the 24-hour incubation. The observed enrichment is therefore consistent with a combination of ongoing cellular activity, stress responses, and subsequent release of intracellular components. Although normalization or compositional effects cannot be fully excluded, total protein concentrations were largely unchanged, and the observed changes were consistent with previous reports and orthogonal validation (e.g., IL-8 by ELISA), supporting a biological rather than technical explanation. In contrast, CSF remained more stable under these conditions, likely because it is an essentially cell-free matrix less susceptible to enzymatic and cellular release effects. This behavior contrasts with freeze–thaw effects, which are primarily driven by physical disruption. Together, these findings indicate that delayed processing of whole blood can introduce structured, non-random changes driven by ex vivo cellular activity, and thus represents a critical pre-analytical variable.

### CSF: centrifugation conditions

Centrifugation speed and conditions can influence the molecular composition of CSF. Higher centrifugation speeds (2000 g) have been associated with lower CSF DJ-1 concentrations compared to those at lower speeds or no centrifugation [32]. In contrast, some studies report that centrifugation speed does not significantly affect Alzheimer’s disease biomarkers in CSF [33]. Similarly, in blood samples, centrifugation conditions have been shown to have a minimal impact on proteomic profiles [34–36]. Consistent with these findings, our study showed no analytes met the predefined statistical and fold-change criteria across the centrifugation speeds evaluated for CSF. These results suggest that routine centrifugation protocols do not substantially alter the CSF protein composition.

### CSF: long-term storage tubes

Low-protein binding treatment of storage tubes has been reported to affect the measurement of CSF biomarkers, particularly in Alzheimer’s disease research [31, 37]. In our study, although some analytes showed a decrease, most remained unaffected by the low-protein binding treatment. As our samples were stored for approximately five years, prolonged storage may have facilitated protein adsorption, potentially contributing to protein reductions even in low-protein binding tubes.

### Plasma: storage tubes

The impact of storage container materials, such as glass and plastic, on blood sample analytes has been reported [38, 39]. However, studies specifically investigating differences in the blood proteome because of variations among manufacturers using the same material are limited. In our study, only minimal differences were observed between polypropylene tubes from different manufacturers, suggesting that manufacturer-related variability has little impact on blood proteomic analysis under the conditions examined in this study.

### CSF: storage temperature

Storage temperature also significantly affects the stability of CSF analytes. Cystatin C undergoes cleavage at -20 °C but remains intact at -80 °C, emphasizing the importance of lower temperatures for proteomic studies [40]. Similarly, amyloid-β1–42 and tau concentrations remain stable at -80 °C, whereas significant degradation occurs at higher temperatures [41]. In line with these findings, our study also demonstrated substantial molecular alterations between storage at -80 °C and − 20 °C, highlighting the critical role of temperature selection for medium- to long-term CSF preservation.

### Plasma: storage temperature

In our study, we observed extensive molecular changes in samples stored at -20 °C when compared with those stored at -80 °C, indicating a substantial impact of higher storage temperatures on plasma composition. These findings are consistent with prior proteomic studies demonstrating that − 80 °C is optimal for preserving the plasma proteome, whereas storage at − 20 °C induces significant changes in protein abundance, including major plasma proteins such as albumin and fibrinogen [42]. Notably, our data further suggest that proteomic profiles at − 50 °C are largely comparable to those at − 80 °C, indicating a potential threshold below which protein stability is preserved.

### CSF: freeze-thaw cycles

Repeated freeze-thaw cycles can affect CSF analytes in a cycle-dependent manner, potentially altering biomarker concentrations and leading to misinterpretation of results. Several studies have demonstrated that repeated freeze-thaw cycles can cause irregular elevations in CSF GABA levels [43] and influence tau and phosphorylated tau concentrations [29]. In our study, extensive freeze–thaw cycling (11 cycles, including an additional 10 cycles of thawing and refreezing) resulted in pronounced changes in multiple analytes, whereas only limited effects were observed after two cycles, with changes remaining modest even after three cycles. These findings support, under the conditions examined in this study, the practical use of surplus samples and more flexible aliquoting strategies, such as preparing larger initial aliquots that can be subdivided as needed.

### Plasma: freeze-thaw cycles

Regarding the impact of freeze–thaw cycles, previous studies have demonstrated that up to 3–4 cycles have minimal effects on the stability of most plasma proteins, especially when analyzed at the peptide level [44]. Mass spectrometry analyses have even shown high resistance up to 25 cycles [44]. Our results align with these findings, showing very few altered analytes up to three cycles. However, investigators should be aware that specific sensitive molecules, such as ceruloplasmin (CP) and IGHV6-1, may exhibit significant changes after just a single freeze–thaw cycle [45]. The GO enrichment analyses provide mechanistic insight into freeze–thaw–induced proteomic changes. In CSF, enrichment of glycosylation-related processes suggests that glycoproteins may be particularly susceptible to structural alterations during freeze–thaw cycles. In plasma, enrichment of vesicle transport–related proteins, particularly among those showing increased abundance, is consistent with disruption of extracellular vesicles or vesicle-associated protein complexes, potentially leading to the release or altered detectability of vesicle-associated proteins. In addition, analyses restricted to proteins showing increased abundance after freeze–thaw cycles further supported enrichment of vesicle-related and intracellular transport processes, reinforcing the interpretation that physical disruption contributes to the observed changes. Overall, these findings suggest that freeze–thaw effects are not random but reflect selective vulnerability of specific molecular features, particularly glycoproteins in CSF and vesicle-associated proteins in plasma. Plasma contains abundant proteases, coagulation factors, and cellular components, which may promote protein degradation or the release of intracellular proteins during sample handling. In contrast, CSF is an essentially cell-free, low-protein matrix, which may contribute to its apparent stability under certain pre-analytical conditions. However, the lower protein concentration in CSF may reduce buffering capacity and increase susceptibility to physical stress, such as freeze–thaw cycles, particularly in low-protein environments [46]. These differences suggest that distinct mechanisms underlie pre-analytical variability in CSF and plasma, with plasma being more susceptible to enzymatic effects and CSF to physical stress.

### Significance and limitations of this study

The findings of this study provide exploratory insights into the impact of common pre-analytical variables on CSF and plasma proteomes and may inform the evaluation of processing conditions in biobanks and multi-center collaborative studies. However, several limitations should be acknowledged. First, the sample size was limited (*n* = 3–4 per condition), which restricts statistical power and may lead to underestimation of pre-analytical effects. Consequently, the absence of statistically significant changes for most analytes under certain conditions should not be interpreted as evidence that no effect exists. Rather, small but potentially biologically meaningful effects may not have been detected and could become apparent in studies with larger sample sizes and independent validation cohorts. Therefore, caution is warranted when generalizing these findings, and validation in larger, independent cohorts will be important. Accordingly, this study should be regarded as exploratory and hypothesis-generating. Second, although differential statistical testing was complemented by fold-change–based criteria, statistical significance alone may not fully capture subtle systematic shifts in protein abundance. To support transparent interpretation, we therefore included supplementary visualizations (e.g., volcano plots and fold-change distributions) for conditions in which few or no analytes met the predefined criteria. These visualizations provide contextual information but do not substitute for adequately powered validation studies. Third, proteomic profiling was performed using the SomaScan platform. While SomaScan offers broad protein coverage with high analytical precision, it relies on affinity-based measurements and may have lower protein specificity compared with antibody-based platforms such as Olink. In addition, although the SomaScan platform employs multiple sample dilutions to ensure that measurements fall within validated linear ranges, analyte-specific information on dilution selection, linearity limits, and dynamic ranges is not disclosed to end users. As a result, analyte-level verification of linearity is not possible. To mitigate this limitation, we restricted downstream analyses to analytes that passed SomaLogic’s quality control criteria and exhibited high analytical reproducibility (CV < 15% based on repeated measurements). Nevertheless, reported fold-change values should be interpreted with appropriate caution, particularly for individual analytes. Validation using orthogonal proteomic platforms, as well as integration with other omics approaches (e.g., metabolomics), would further strengthen the generalizability of the findings.

### Implications for sample processing and biomarker discovery

**Fig. 4:**
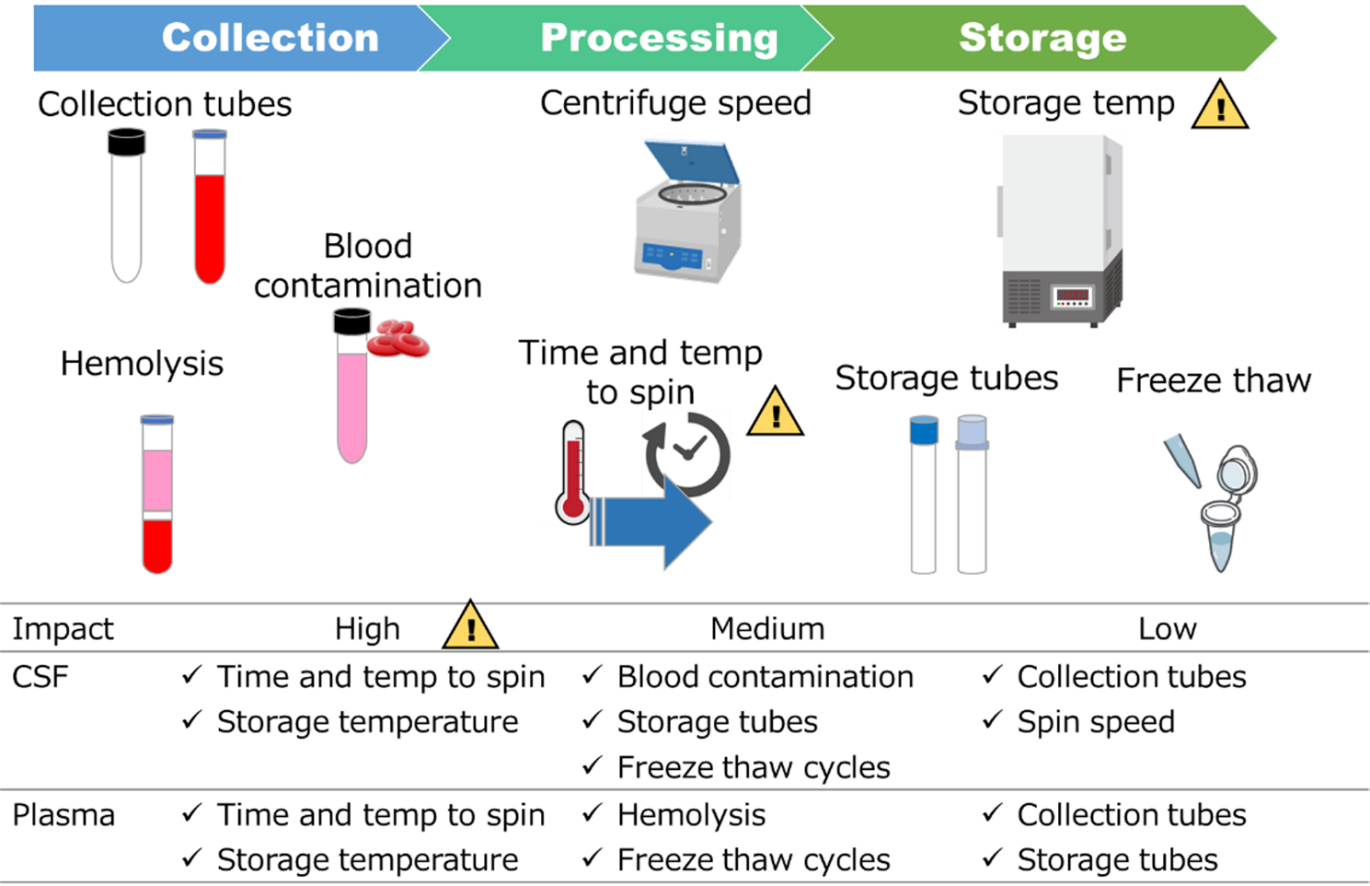
Schematic overview of pre-analytical variables (PAVs) and their impact on biomarker discovery. Pre-analytical factors across sample collection, processing, and storage introduce variable and non-random effects on proteomic measurements. Variables such as time and temperature before processing and storage temperature have a broad impact on protein abundance, whereas blood contamination and freeze–thaw cycles affect more specific subsets of proteins. These PAV-specific effects may introduce bias in biomarker discovery pipelines, including false positives, false negatives, and reduced reproducibility

Based on our analyses, several practical considerations for sample handling can be proposed. For CSF, when processing is delayed beyond 4 h, storage at 4 °C is preferable, and storage at − 80 °C minimizes proteomic changes compared to − 20 °C. For plasma, processing within 2 h limits variability, and storage at − 50 °C yields profiles comparable to − 80 °C. In both matrices, up to three freeze–thaw cycles had minimal impact, and blood contamination showed limited overall effects. Delayed processing and elevated temperatures affected a broad range of proteins, including inflammatory mediators such as IL-8, potentially mimicking disease-associated changes. In contrast, blood contamination selectively increased a limited number of abundant blood-derived proteins, posing a risk of false-positive findings. These results suggest that biomarkers differ in their sensitivity to pre-analytical conditions, which may bias discovery-phase studies and impair reproducibility across cohorts. In studies using existing samples, where pre-analytical variability is unavoidable, our findings provide a framework for interpreting proteomic data and prioritizing candidate biomarkers. Incorporating pre-analytical sensitivity into biomarker discovery may reduce false positives and improve robustness of validation. Overall, these results highlight the importance of integrating pre-analytical considerations into study design and data interpretation (Fig. 4).

## Supplementary Information

Below is the link to the electronic supplementary material.

Supplementary Material 1: Figure S1. Criteria for Defining Changes in Analyte Levels. Volcano plots illustrate the criteria used to define analyte changes based on fold change and statistical significance. In both panels, the horizontal line indicates − log₁₀ (p) = 1.3, corresponding to a nominal p-value of 0.05. Left panel: Analytes with fold changes > 20.5 or < 2− 0.5 and nominal p < 0.05 are highlighted in yellow and were defined as changed analytes. Right panel: Analytes with fold changes > 2 or < 0.5 and nominal p < 0.05 are highlighted in red and were defined as analytes with large changes, representing a subset of the changed analytes.

Supplementary Material 2: Figure S2. Impact of blood contamination on the CSF proteome analyzed by volcano plots. Cerebrospinal fluid (CSF) samples without blood contamination were spiked with autologous whole blood to achieve red blood cell (RBC) concentrations of 100, 500, and 5000 cells/mm³. Volcano plots were generated to compare each blood-spiked condition with the corresponding unspiked control. The y-axis represents the negative logarithm of the p-value from the t-test, and the x-axis indicates the logarithm of the fold change between the two conditions. A: Volcano plot comparing CSF samples without blood contamination and those spiked to an RBC concentration of 100 cells/mm³. B: Volcano plot comparing CSF samples without blood contamination and those spiked to an RBC concentration of 500 cells/mm³. C: Volcano plot comparing CSF samples without blood contamination and those spiked to an RBC concentration of 5000 cells/mm³.

Supplementary Material 3: Figure S3. Examples of proteins increased by blood contamination in CSF. Cerebrospinal fluid (CSF) samples without blood contamination were spiked with autologous whole blood to achieve red blood cell (RBC) concentrations of 100, 500, and 5000 cells/mm³. Fold changes relative to the unspiked control were calculated for selected analytes. **A**: LL-37 measured by SomaScan, representing the analyte with the largest relative increase. **B**: Fibrinogen measured by SomaScan. **C**: Fibrinogen measured by ELISA. Data are presented as mean ± standard deviation.

Supplementary Material 4: Figure S4. Distribution of coefficients of variation (CVs) across blood contamination levels. Cerebrospinal fluid (CSF) samples without blood contamination were spiked with autologous whole blood to achieve red blood cell (RBC) concentrations of 100, 500, and 5000 cells/mm³. Coefficients of variation (CVs) were calculated for each analyte based on repeated measurements of pooled samples. Violin plots were generated to visualize the distribution of CV values across the different blood contamination levels. The width of each violin represents the density of analytes at a given CV value.

Supplementary Material 5: Figure S5. Impact of collection tubes on CSF and plasma proteomes analyzed by volcano plots. Volcano plots were generated to assess the impact of different collection containers on cerebrospinal fluid (CSF) and plasma proteomes. Axes and statistical analyses are as described in Figures S2. **A**: Volcano plot comparing the CSF proteome collected using a polypropylene tube with that collected using a low protein-binding tube (baseline condition). **B**: Volcano plot comparing the CSF proteome collected using a polystyrene tube with that collected using a low protein-binding tube (baseline condition). **C**: Volcano plot comparing the plasma proteome collected using an Insepack tube (Sekisui Medical) with that collected using a Venoject tube (Terumo; baseline condition).

Supplementary Material 6: Figure S6. Impact of hemolysis on the plasma proteome analyzed by volcano plot. Artificial hemolysis was induced by vortexing plasma samples for approximately 2 minutes and compared with non-hemolyzed control samples. A volcano plot was generated to assess differences between hemolyzed and non-hemolyzed conditions. Axes and statistical analyses are as described in Figures S2.

Supplementary Material 7: Figure S7. Impact of time and temperature before processing on the CSF proteome analyzed by volcano plots. After collection, CSF samples were left to stand at either 4°C or 25°C for 0, 2, 4, or 24 hours prior to processing. Volcano plots were generated to compare each delayed-processing condition with the baseline condition processed immediately at 4°C (0 h). Axes and statistical analyses are as described in Figures S2.

Supplementary Material 8: Figure S8. Impact of time and temperature before processing on the plasma proteome analyzed by volcano plots. After blood collection, plasma samples were kept at either 4°C (for up to 72 h) or 25°C (for up to 24 h) for defined periods prior to processing. Volcano plots were generated to compare each delayed-processing condition with the baseline condition processed after 30 minutes at 4°C. Axes and statistical analyses are as described in Figures S2. (**A**–**C**) Plasma samples kept at 4°C for 2, 24, or 72 h before processing. (**D**–**E**) Plasma samples kept at 25°C for 2 or 24 h before processing.

Supplementary Material s9: Table s9. CSF Freeze thaw cycles: analytes showing large changes

Supplementary Material 9: Figure S9. GO enrichment analysis of plasma proteins altered after 24 h incubation at 25°C. Plasma samples were incubated at 25°C for 24 hours prior to processing and compared with the corresponding baseline condition. Gene Ontology (GO) enrichment analysis was performed on proteins that showed large change. The dot plot displays enriched biological processes, with the x-axis representing the gene ratio (enrichment strength) and the y-axis indicating GO terms. Dot size corresponds to the number of proteins associated with each term, and color indicates the adjusted p-value (Benjamini–Hochberg correction).

Supplementary Material 10: Figure S10. Comparison of IL-8 measurements between SomaScan and ELISA under delayed processing conditions. Plasma samples were incubated at 25°C for 24 hours prior to centrifugation and compared with the corresponding baseline condition. IL-8 concentrations were measured using both SomaScan (right panel) and ELISA (left panel). Paired measurements from the same samples are connected by lines, illustrating changes between baseline (“RT 0.5hr”) and delayed processing (“RT 24hr”) conditions. Because one baseline ELISA measurement was below the limit of detection, fold change could not be calculated for all samples. Therefore, results are presented as paired plots rather than fold changes to allow inclusion of all data points. Despite differences in measurement scale between platforms, consistent directional increases in IL-8 were observed across samples. (**A**–**C**) CSF samples kept at 4°C for 2, 4, or 24 h before processing. (**D**–**F**) CSF samples kept at 25°C for 2, 4, or 24 h before processing.

Supplementary Material 11: Figure S11. Impact of centrifugation conditions on the CSF proteome analyzed by volcano plots. CSF samples were centrifuged at 1000 g, 2000 g, or 4000 g for 10 minutes, and the resulting supernatants were stored for analysis. Volcano plots were generated to compare each centrifugation condition with the baseline condition of 2000 g. Axes and statistical analyses are as described in Figures S2. **A**: Volcano plot comparing CSF samples centrifuged at 1000 g with those centrifuged at 2000 g. **B**: Volcano plot comparing CSF samples centrifuged at 4000 g with those centrifuged at 2000 g.

Supplementary Material 12: Figure S12. Impact of storage tubes on CSF and plasma proteomes analyzed by volcano plots. Volcano plots were generated to assess the impact of different storage tubes on cerebrospinal fluid (CSF) and plasma proteomes. Axes and statistical analyses are as described in Figures S2. **A**: CSF samples processed under identical conditions were aliquoted into polypropylene tubes (96 Jacket tubes, FCR & Bio) or low protein-binding tubes (PROTEOSAVE SS 1.5 mL Slimtube, Sumitomo Bakelite). The volcano plot compares CSF samples aliquoted into polypropylene tubes with those aliquoted into low protein-binding tubes (baseline condition). **B**: After centrifugation, plasma samples were aliquoted into 96 Jacket tubes (FCR & Bio) or Matrix tubes (Thermo Fisher Japan). The volcano plot compares plasma samples aliquoted into Matrix tubes with those aliquoted into 96 Jacket tubes (baseline condition).

Supplementary Material 13: Figure S13. Impact of freeze–thaw cycles on CSF and plasma proteomes analyzed by volcano plots. Aliquoted CSF and plasma samples were subjected to 1, 2, 3, or 11 freeze–thaw cycles prior to analysis. Volcano plots were generated to compare each condition with the baseline condition subjected to a single freeze–thaw cycle. Axes and statistical analyses are as described in Figures S2. **A**: Volcano plots comparing CSF samples subjected to 2, 3, or 11 freeze–thaw cycles with those subjected to one cycle (reference condition). **B**: Volcano plots comparing plasma samples subjected to 2, 3, or 11 freeze–thaw cycles with those subjected to one cycle (reference condition).

Supplementary Material 14: Figure S14. GO enrichment analysis of CSF proteins altered after 11 freeze–thaw cycles. Cerebrospinal fluid (CSF) samples were subjected to 11 freeze–thaw cycles and compared with samples subjected to a single freeze–thaw cycle (baseline condition). Gene Ontology (GO) enrichment analysis was performed on proteins that showed large change. The dot plot displays enriched biological processes, with the x-axis representing the gene ratio (enrichment strength) and the y-axis indicating GO terms. Dot size corresponds to the number of proteins associated with each term, and color indicates the adjusted p-value (Benjamini–Hochberg correction).

Supplementary Material 15: Figure S15. GO enrichment analysis of plasma proteins altered after 11 freeze–thaw cycles. Plasma samples were subjected to 11 freeze–thaw cycles and compared with samples subjected to a single freeze–thaw cycle (baseline condition). Gene Ontology (GO) enrichment analysis was performed on proteins that showed large change. **A**: GO enrichment analysis including all significantly altered proteins. **B**: GO enrichment analysis restricted to proteins showing increased abundance. The dot plots display enriched biological processes, with the x-axis representing the gene ratio (enrichment strength) and the y-axis indicating GO terms. Dot size corresponds to the number of proteins associated with each term, and color indicates the adjusted p-value (Benjamini–Hochberg correction).

Supplementary Material 16: Table S1. CSF blood contamination: top 10 high-abundance blood proteins selected from the Human Protein Atlas and their SomaScan measurements.

Supplementary Material 17: Table S2. CSF blood contamination: analytes showing large changes.

Supplementary Material 18: Table S3. Plasma hemolysis: analytes showing large changes.

Supplementary Material 19: Table S4. CSF pre-processing (time & temperature): analytes showing large changes.

Supplementary Material 20: Table S5. Plasma pre-processing (time & temperature): analytes showing large changes.

Supplementary Material 21: Table S6. Gene Ontology (GO) enrichment analysis of proteins altered after 24 h incubation at 25°C in plasma.

Supplementary Material 22: Table S7. CSF storage temperature (-80°C vs -20°C): analytes showing large changes.

Supplementary Material 23: Table S8. Plasma storage temperature (-80°C, -50°C, -20°C): analytes showing large changes.

Supplementary Material 24: Table S10. Plasma Freeze thaw cycles: analytes showing large changes.

Supplementary Material 25: Table S11. Gene Ontology (GO) enrichment analysis of proteins altered after 11 freeze–thaw cycles in CSF.

Supplementary Material 26: Table S12. Gene Ontology (GO) enrichment analysis of proteins altered after 11 freeze–thaw cycles in plasma.

Supplementary Material 27: Table S13. GO enrichment analysis of proteins with increased abundance after 11 freeze–thaw cycles in plasma

## Data Availability

The datasets supporting this study have been deposited in the PRIDE repository (accession number PAD000011) and are currently under embargo in accordance with a consortium agreement. They will be publicly available after March 31, 2028.

## Abbreviations

CSF: Cerebrospinal fluid
CV: Coefficient of variation
NfL: Neurofilament light chain
PAV: Pre-analytical variable
p-Tau: Phosphorylated tau
RBC: Red blood cell
