# Supplementary figures and images for: The effect of preanalytical factors on cerebrospinal fluid and plasma proteomics: a systematic experimental study
Source: Clin Proteomics. 2026 May 22;23:40. doi: 10.1186/s12014-026-09604-5 (PMC13383461; doi:10.1186/s12014-026-09604-5)

## Slide 1
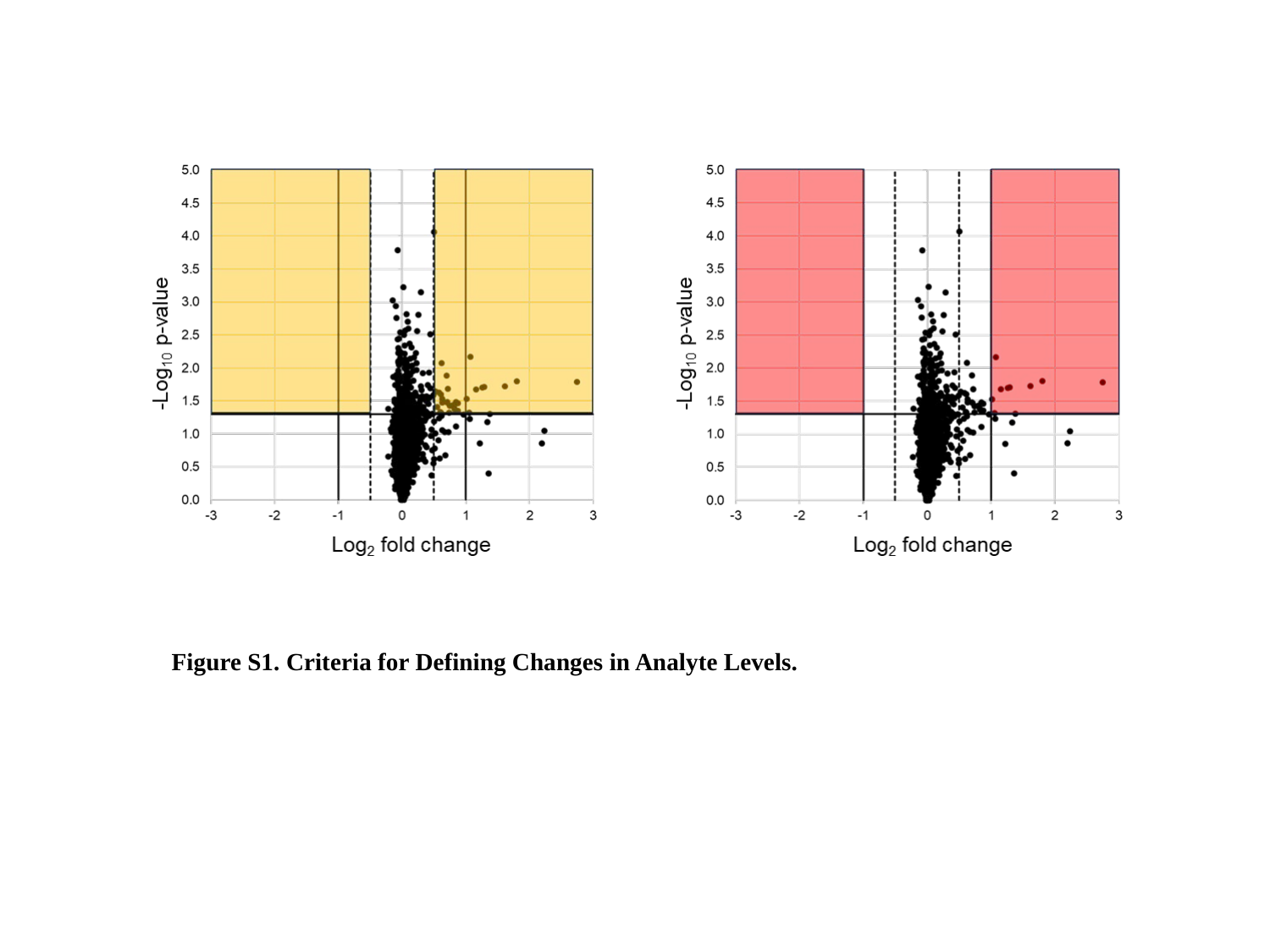

Figure S1. Criteria for Defining Changes in Analyte Levels.

Supplement: Supplementary file 1 — Supplementary Material 1: Figure S1. Criteria for Defining Changes in Analyte Levels. Volcano plots illustrate the criteria used to define analyte changes based on fold change and statistical significance. In both panels, the horizontal line indicates − log₁₀ (p) = 1.3, corresponding to a nominal p-value of 0.05. Left panel: Analytes with fold changes > 20.5 or < 2− 0.5 and nominal p < 0.05 are highlighted in yellow and were defined as changed analytes. Right panel: Analytes with fold changes > 2 or < 0.5 and nominal p < 0.05 are highlighted in red and were defined as analytes with large changes, representing a subset of the changed analytes. [file 12014_2026_9604_MOESM1_ESM.pptx]

## Slide 1
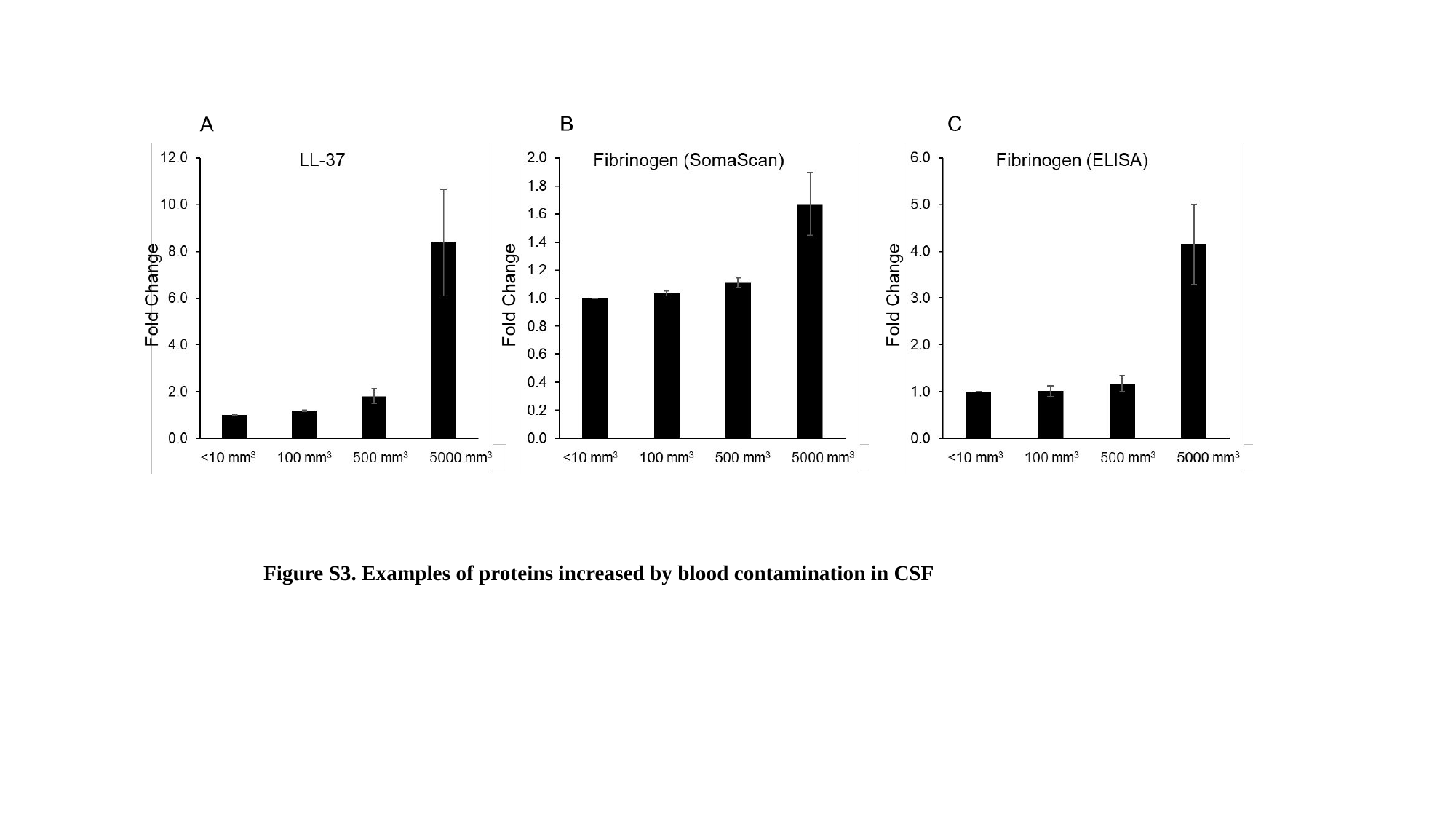

Figure S3. Examples of proteins increased by blood contamination in CSF

Supplement: Supplementary file 3 — Supplementary Material 3: Figure S3. Examples of proteins increased by blood contamination in CSF. Cerebrospinal fluid (CSF) samples without blood contamination were spiked with autologous whole blood to achieve red blood cell (RBC) concentrations of 100, 500, and 5000 cells/mm³. Fold changes relative to the unspiked control were calculated for selected analytes. A: LL-37 measured by SomaScan, representing the analyte with the largest relative increase. B: Fibrinogen measured by SomaScan. C: Fibrinogen measured by ELISA. Data are presented as mean ± standard deviation. [file 12014_2026_9604_MOESM3_ESM.pptx]

## Slide 1
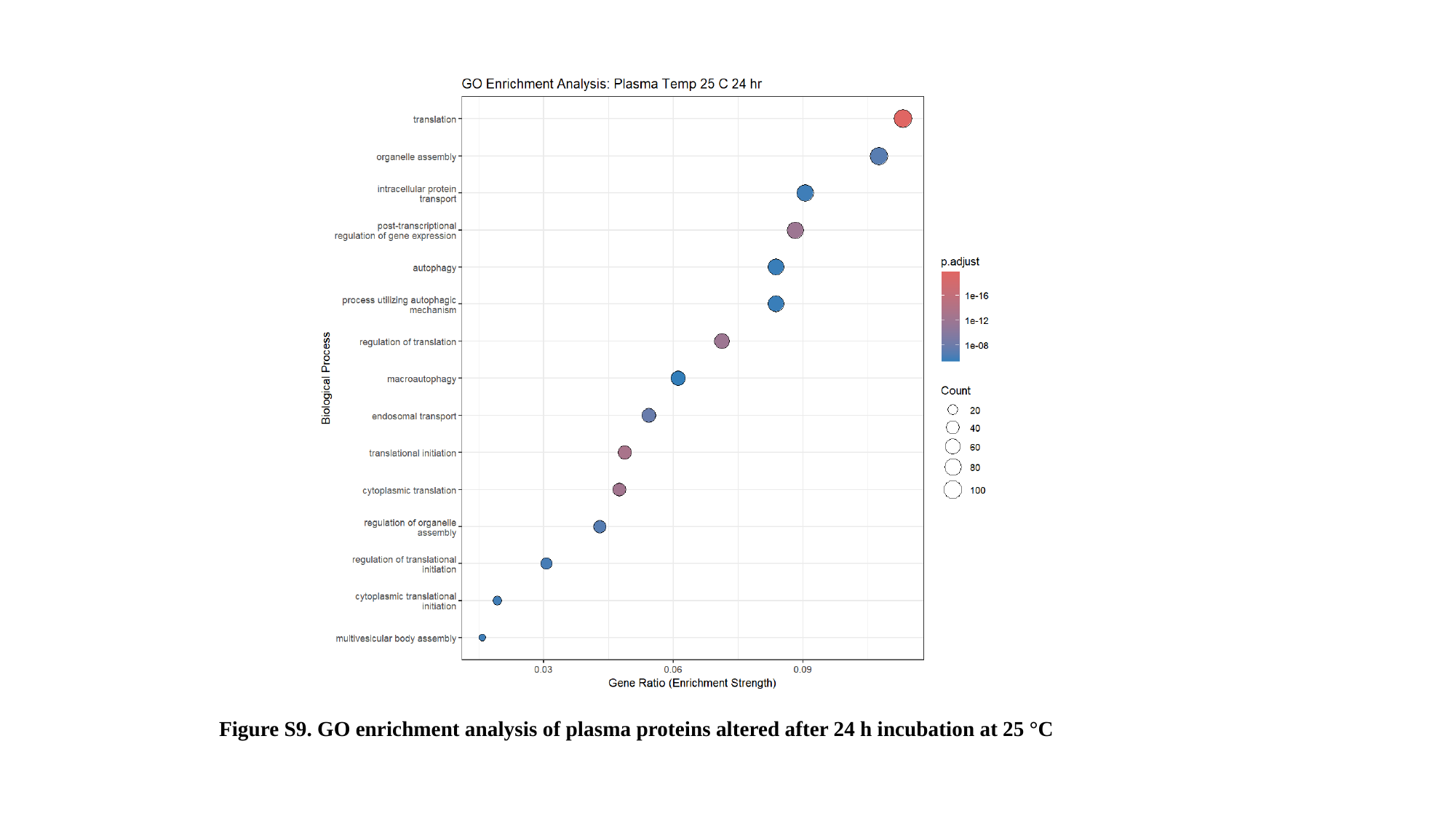

Figure S9. GO enrichment analysis of plasma proteins altered after 24 h incubation at 25 °C

Supplement: Supplementary file 9 — Supplementary Material s9: Table s9. CSF Freeze thaw cycles: analytes showing large changes [file 12014_2026_9604_MOESM9_ESM.pptx]

## Slide 1
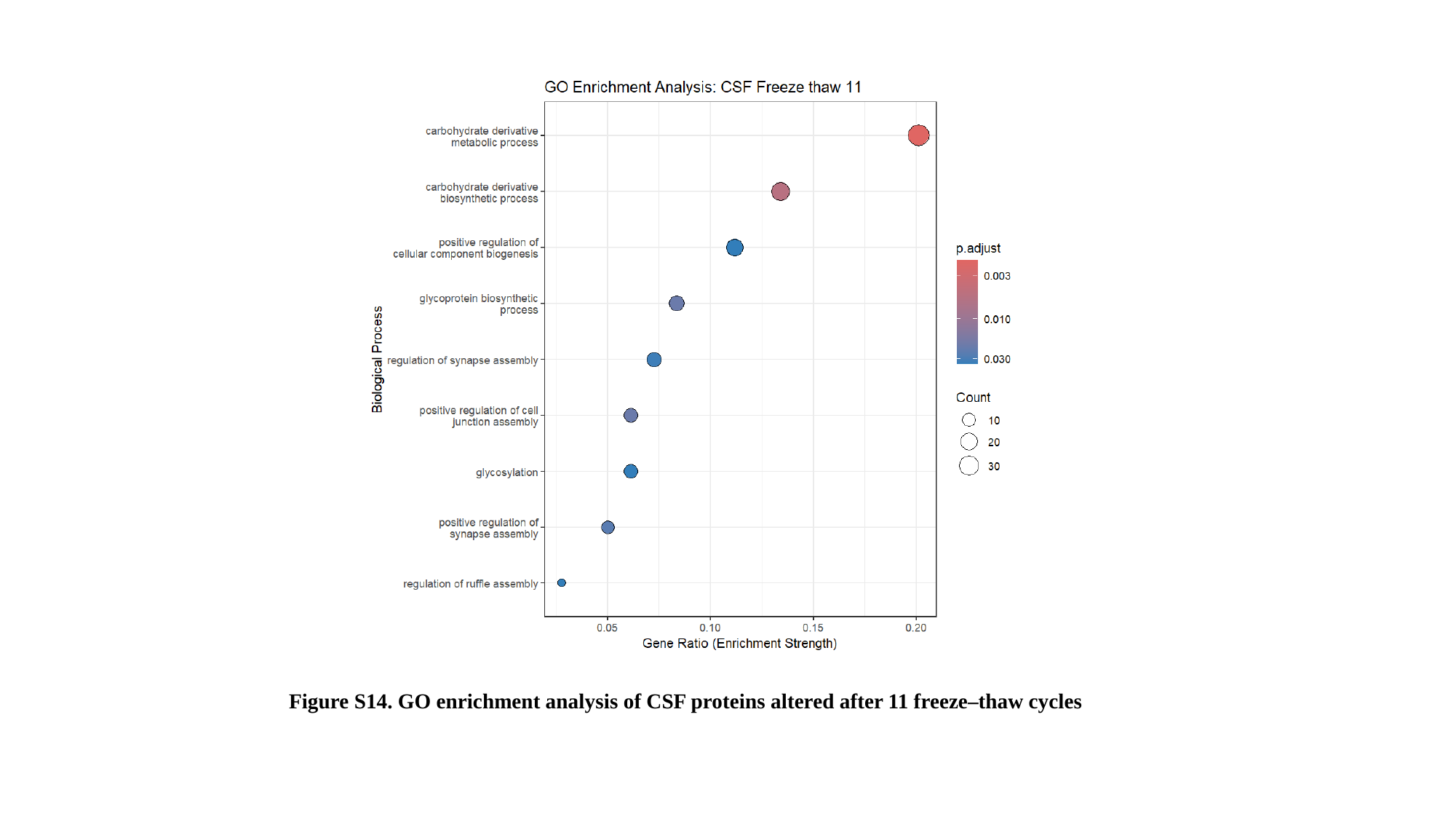

Figure S14. GO enrichment analysis of CSF proteins altered after 11 freeze–thaw cycles

Supplement: Supplementary file 14 — Supplementary Material 13: Figure S13. Impact of freeze–thaw cycles on CSF and plasma proteomes analyzed by volcano plots. Aliquoted CSF and plasma samples were subjected to 1, 2, 3, or 11 freeze–thaw cycles prior to analysis. Volcano plots were generated to compare each condition with the baseline condition subjected to a single freeze–thaw cycle. Axes and statistical analyses are as described in Figures S2. A: Volcano plots comparing CSF samples subjected to 2, 3, or 11 freeze–thaw cycles with those subjected to one cycle (reference condition). B: Volcano plots comparing plasma samples subjected to 2, 3, or 11 freeze–thaw cycles with those subjected to one cycle (reference condition). [file 12014_2026_9604_MOESM14_ESM.pptx]

## Slide 1
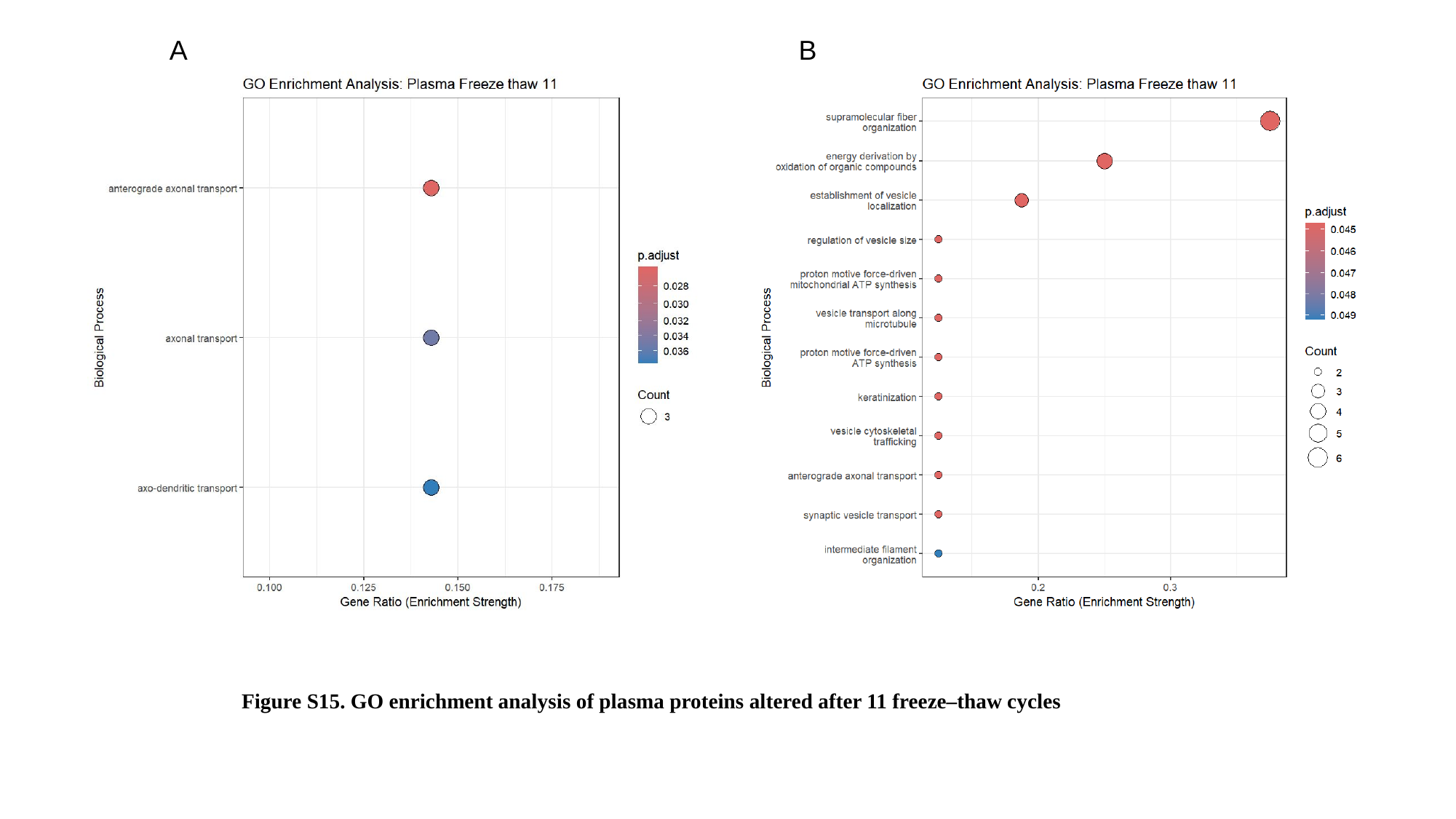

A
B
Figure S15. GO enrichment analysis of plasma proteins altered after 11 freeze–thaw cycles

Supplement: Supplementary file 15 — Supplementary Material 14: Figure S14. GO enrichment analysis of CSF proteins altered after 11 freeze–thaw cycles. Cerebrospinal fluid (CSF) samples were subjected to 11 freeze–thaw cycles and compared with samples subjected to a single freeze–thaw cycle (baseline condition). Gene Ontology (GO) enrichment analysis was performed on proteins that showed large change. The dot plot displays enriched biological processes, with the x-axis representing the gene ratio (enrichment strength) and the y-axis indicating GO terms. Dot size corresponds to the number of proteins associated with each term, and color indicates the adjusted p-value (Benjamini–Hochberg correction). [file 12014_2026_9604_MOESM15_ESM.pptx]
