# Supplementary material for: The effect of preanalytical factors on cerebrospinal fluid and plasma proteomics: a systematic experimental study
Source: Clin Proteomics. 2026 May 22;23:40. doi: 10.1186/s12014-026-09604-5 (PMC13383461; doi:10.1186/s12014-026-09604-5)
Supplement: Supplementary file 2 — Supplementary Material 2: Figure S2. Impact of blood contamination on the CSF proteome analyzed by volcano plots. Cerebrospinal fluid (CSF) samples without blood contamination were spiked with autologous whole blood to achieve red blood cell (RBC) concentrations of 100, 500, and 5000 cells/mm³. Volcano plots were generated to compare each blood-spiked condition with the corresponding unspiked control. The y-axis represents the negative logarithm of the p-value from the t-test, and the x-axis indicates the logarithm of the fold change between the two conditions. A: Volcano plot comparing CSF samples without blood contamination and those spiked to an RBC concentration of 100 cells/mm³. B: Volcano plot comparing CSF samples without blood contamination and those spiked to an RBC concentration of 500 cells/mm³. C: Volcano plot comparing CSF samples without blood contamination and those spiked to an RBC concentration of 5000 cells/mm³. [file 12014_2026_9604_MOESM2_ESM.pptx]

## Slide 1
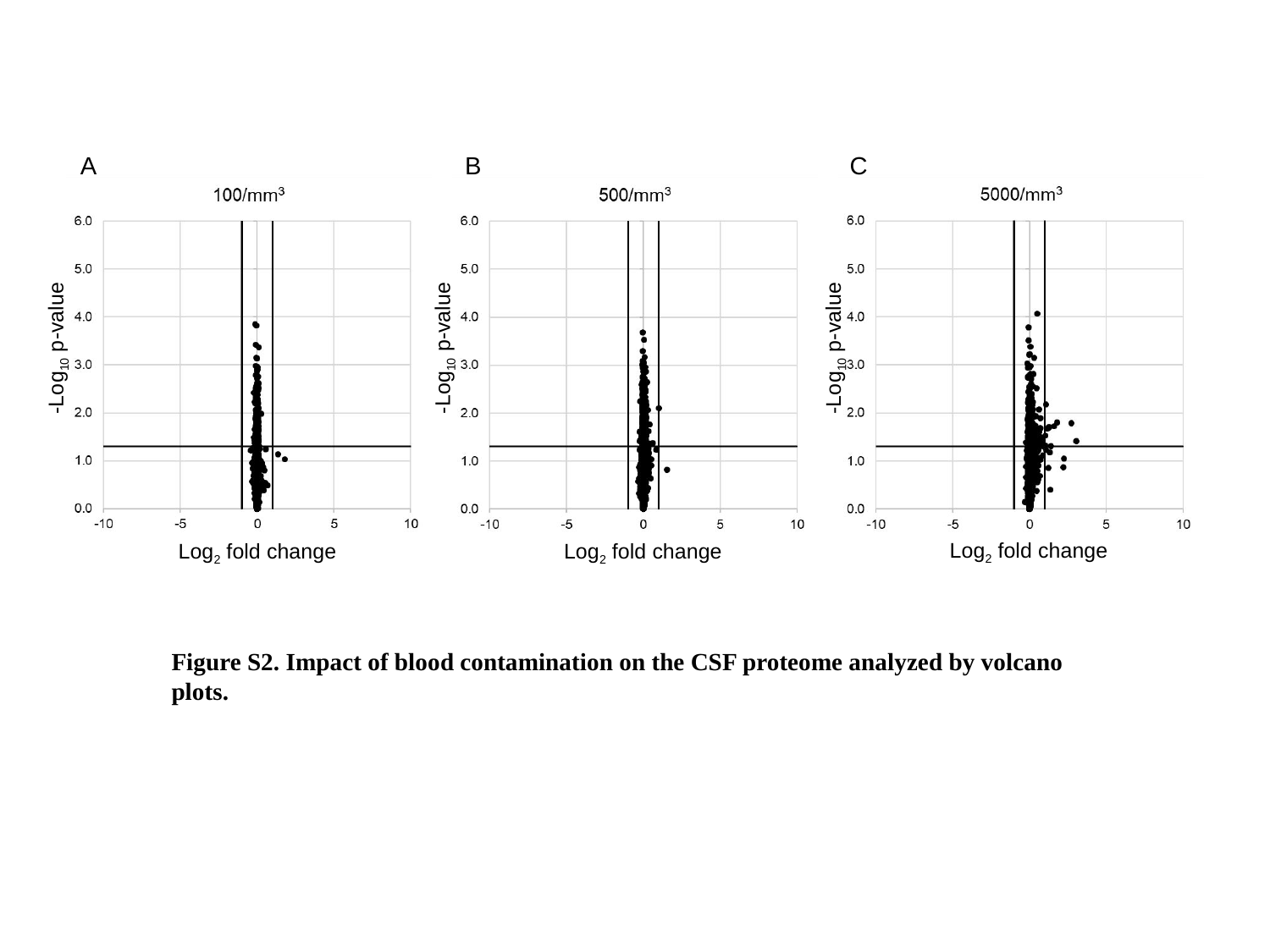

C
B
A
-Log10 p-value
-Log10 p-value
-Log10 p-value
Log2 fold change
Log2 fold change
Log2 fold change
Figure S2. Impact of blood contamination on the CSF proteome analyzed by volcano plots.
