# Supplementary material for: The effect of preanalytical factors on cerebrospinal fluid and plasma proteomics: a systematic experimental study
Source: Clin Proteomics. 2026 May 22;23:40. doi: 10.1186/s12014-026-09604-5 (PMC13383461; doi:10.1186/s12014-026-09604-5)
Supplement: Supplementary file 4 — Supplementary Material 4: Figure S4. Distribution of coefficients of variation (CVs) across blood contamination levels. Cerebrospinal fluid (CSF) samples without blood contamination were spiked with autologous whole blood to achieve red blood cell (RBC) concentrations of 100, 500, and 5000 cells/mm³. Coefficients of variation (CVs) were calculated for each analyte based on repeated measurements of pooled samples. Violin plots were generated to visualize the distribution of CV values across the different blood contamination levels. The width of each violin represents the density of analytes at a given CV value. [file 12014_2026_9604_MOESM4_ESM.pptx]

## Slide 1
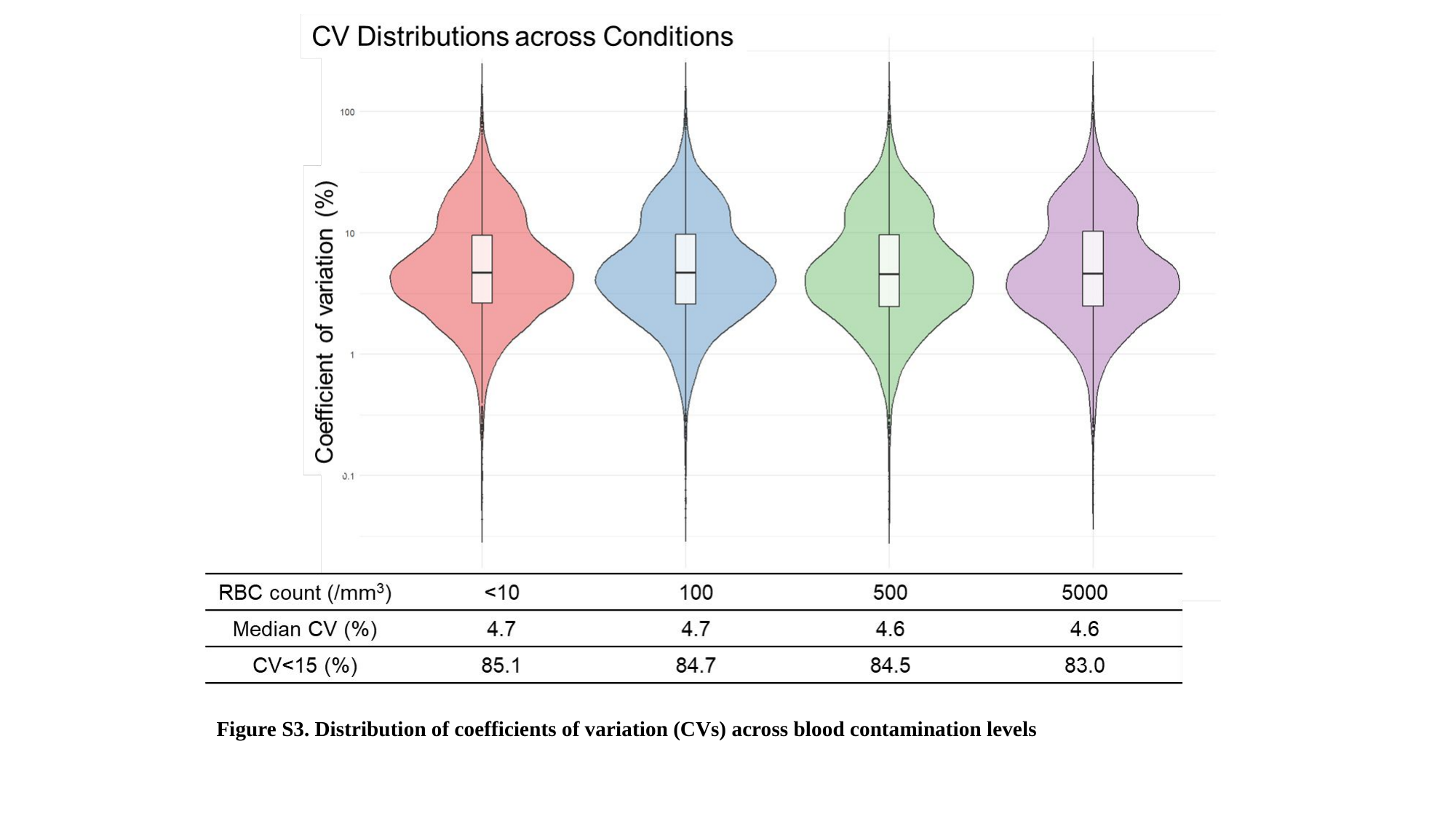

Figure S3. Distribution of coefficients of variation (CVs) across blood contamination levels
