# Supplementary material for: The effect of preanalytical factors on cerebrospinal fluid and plasma proteomics: a systematic experimental study
Source: Clin Proteomics. 2026 May 22;23:40. doi: 10.1186/s12014-026-09604-5 (PMC13383461; doi:10.1186/s12014-026-09604-5)
Supplement: Supplementary file 5 — Supplementary Material 5: Figure S5. Impact of collection tubes on CSF and plasma proteomes analyzed by volcano plots. Volcano plots were generated to assess the impact of different collection containers on cerebrospinal fluid (CSF) and plasma proteomes. Axes and statistical analyses are as described in Figures S2. A: Volcano plot comparing the CSF proteome collected using a polypropylene tube with that collected using a low protein-binding tube (baseline condition). B: Volcano plot comparing the CSF proteome collected using a polystyrene tube with that collected using a low protein-binding tube (baseline condition). C: Volcano plot comparing the plasma proteome collected using an Insepack tube (Sekisui Medical) with that collected using a Venoject tube (Terumo; baseline condition). [file 12014_2026_9604_MOESM5_ESM.pptx]

## Slide 1
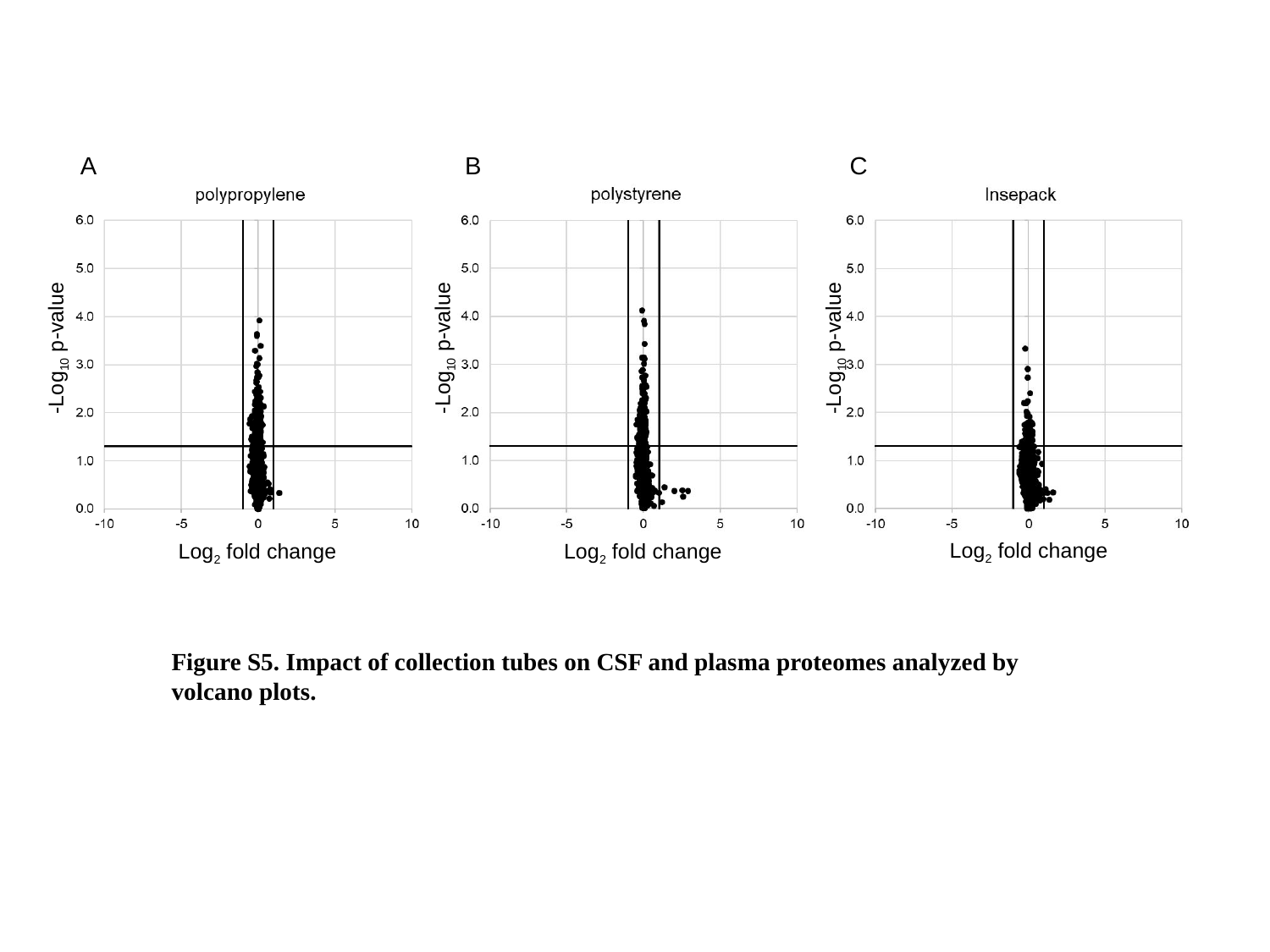

C
B
A
-Log10 p-value
-Log10 p-value
-Log10 p-value
Log2 fold change
Log2 fold change
Log2 fold change
Figure S5. Impact of collection tubes on CSF and plasma proteomes analyzed by volcano plots.
