# Supplementary material for: The effect of preanalytical factors on cerebrospinal fluid and plasma proteomics: a systematic experimental study
Source: Clin Proteomics. 2026 May 22;23:40. doi: 10.1186/s12014-026-09604-5 (PMC13383461; doi:10.1186/s12014-026-09604-5)
Supplement: Supplementary file 6 — Supplementary Material 6: Figure S6. Impact of hemolysis on the plasma proteome analyzed by volcano plot. Artificial hemolysis was induced by vortexing plasma samples for approximately 2 minutes and compared with non-hemolyzed control samples. A volcano plot was generated to assess differences between hemolyzed and non-hemolyzed conditions. Axes and statistical analyses are as described in Figures S2. [file 12014_2026_9604_MOESM6_ESM.pptx]

## Slide 1
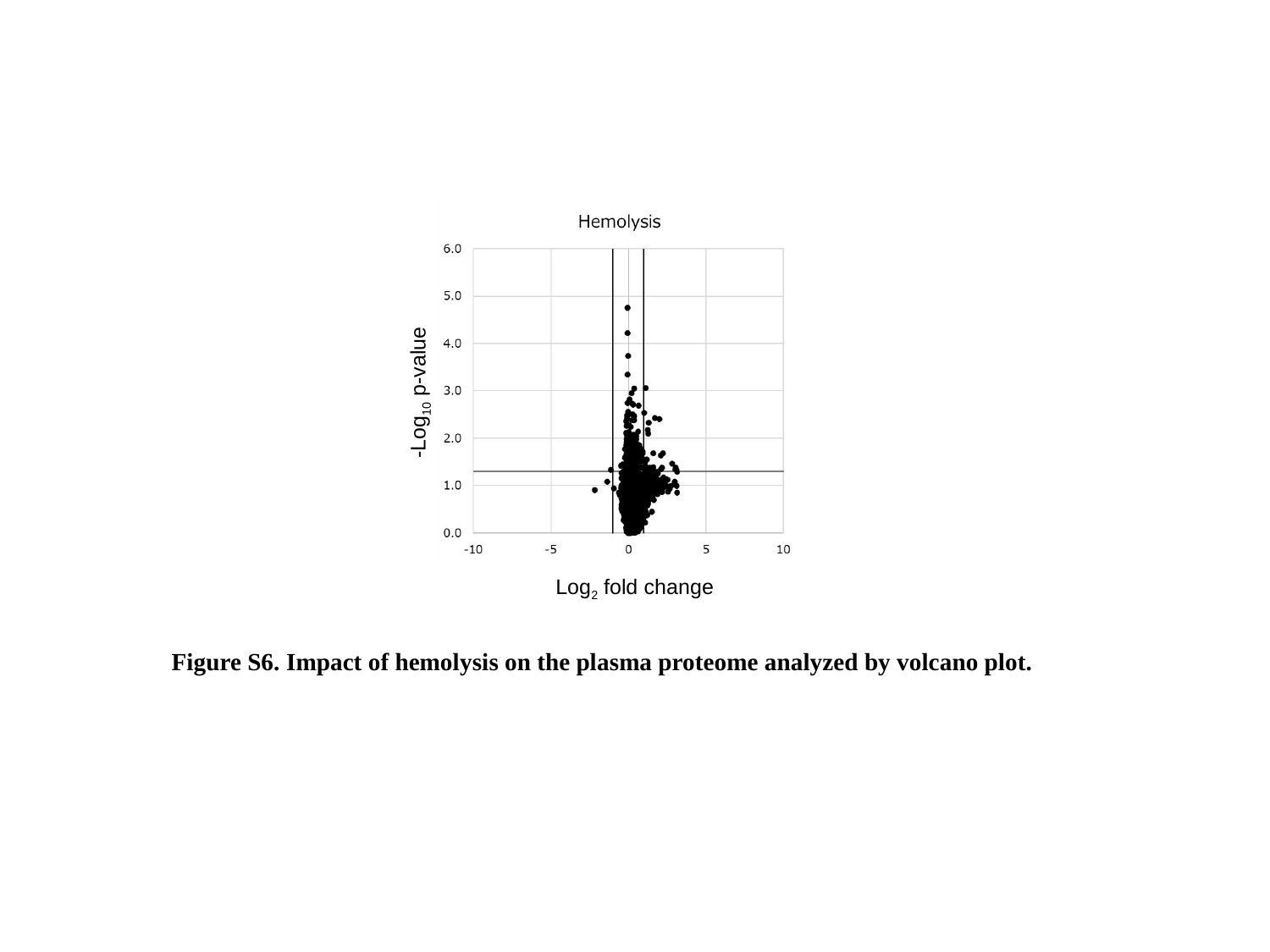

-Log10 p-value
Log2 fold change
Figure S6. Impact of hemolysis on the plasma proteome analyzed by volcano plot.
