# Supplementary material for: The effect of preanalytical factors on cerebrospinal fluid and plasma proteomics: a systematic experimental study
Source: Clin Proteomics. 2026 May 22;23:40. doi: 10.1186/s12014-026-09604-5 (PMC13383461; doi:10.1186/s12014-026-09604-5)
Supplement: Supplementary file 7 — Supplementary Material 7: Figure S7. Impact of time and temperature before processing on the CSF proteome analyzed by volcano plots. After collection, CSF samples were left to stand at either 4°C or 25°C for 0, 2, 4, or 24 hours prior to processing. Volcano plots were generated to compare each delayed-processing condition with the baseline condition processed immediately at 4°C (0 h). Axes and statistical analyses are as described in Figures S2. [file 12014_2026_9604_MOESM7_ESM.pptx]

## Slide 1
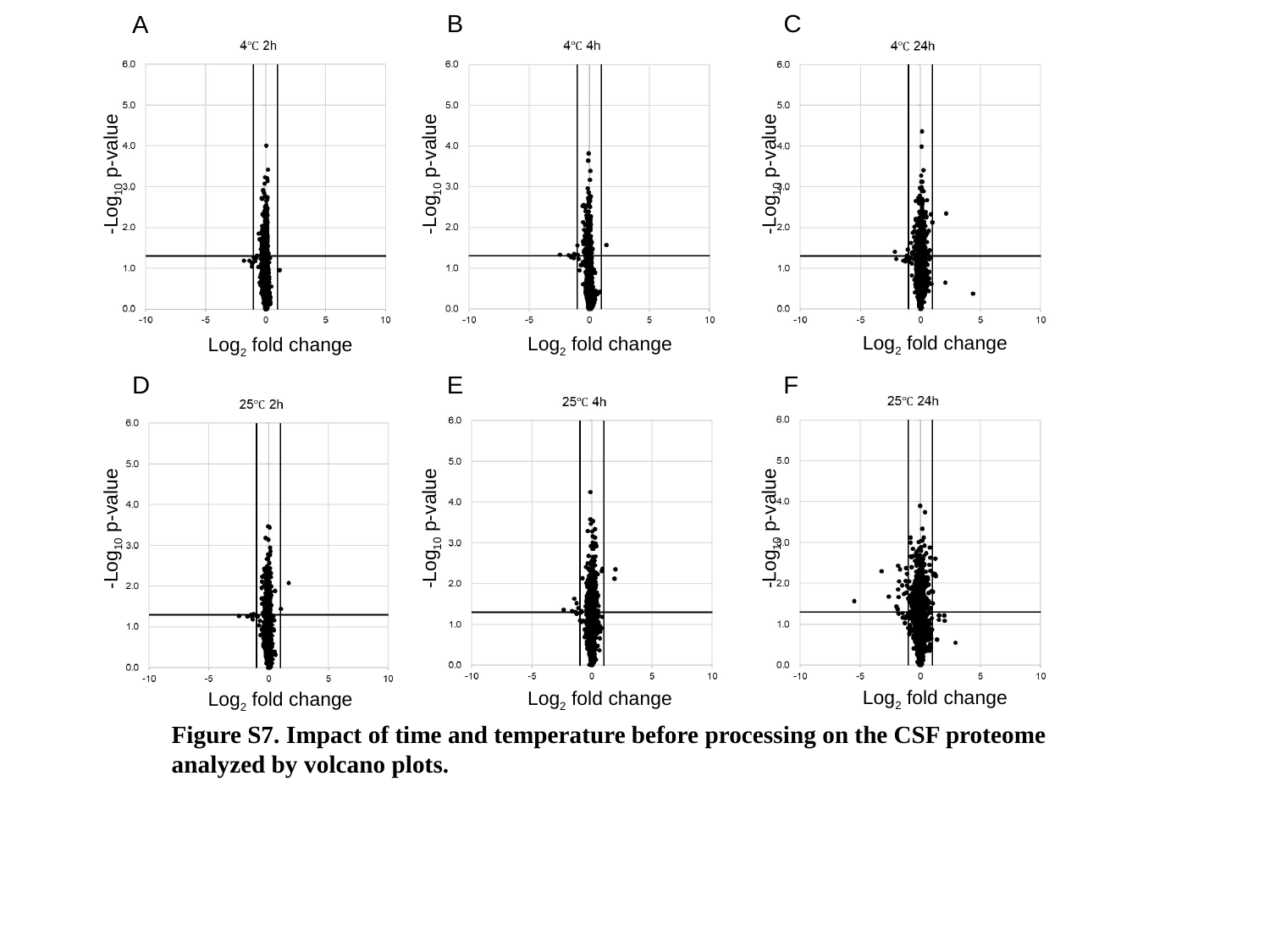

B
C
A
-Log10 p-value
-Log10 p-value
-Log10 p-value
Log2 fold change
Log2 fold change
Log2 fold change
E
F
D
-Log10 p-value
-Log10 p-value
-Log10 p-value
Log2 fold change
Log2 fold change
Log2 fold change
Figure S7. Impact of time and temperature before processing on the CSF proteome analyzed by volcano plots.
