# Supplementary material for: The effect of preanalytical factors on cerebrospinal fluid and plasma proteomics: a systematic experimental study
Source: Clin Proteomics. 2026 May 22;23:40. doi: 10.1186/s12014-026-09604-5 (PMC13383461; doi:10.1186/s12014-026-09604-5)
Supplement: Supplementary file 8 — Supplementary Material 8: Figure S8. Impact of time and temperature before processing on the plasma proteome analyzed by volcano plots. After blood collection, plasma samples were kept at either 4°C (for up to 72 h) or 25°C (for up to 24 h) for defined periods prior to processing. Volcano plots were generated to compare each delayed-processing condition with the baseline condition processed after 30 minutes at 4°C. Axes and statistical analyses are as described in Figures S2. (A–C) Plasma samples kept at 4°C for 2, 24, or 72 h before processing. (D–E) Plasma samples kept at 25°C for 2 or 24 h before processing. [file 12014_2026_9604_MOESM8_ESM.pptx]

## Slide 1
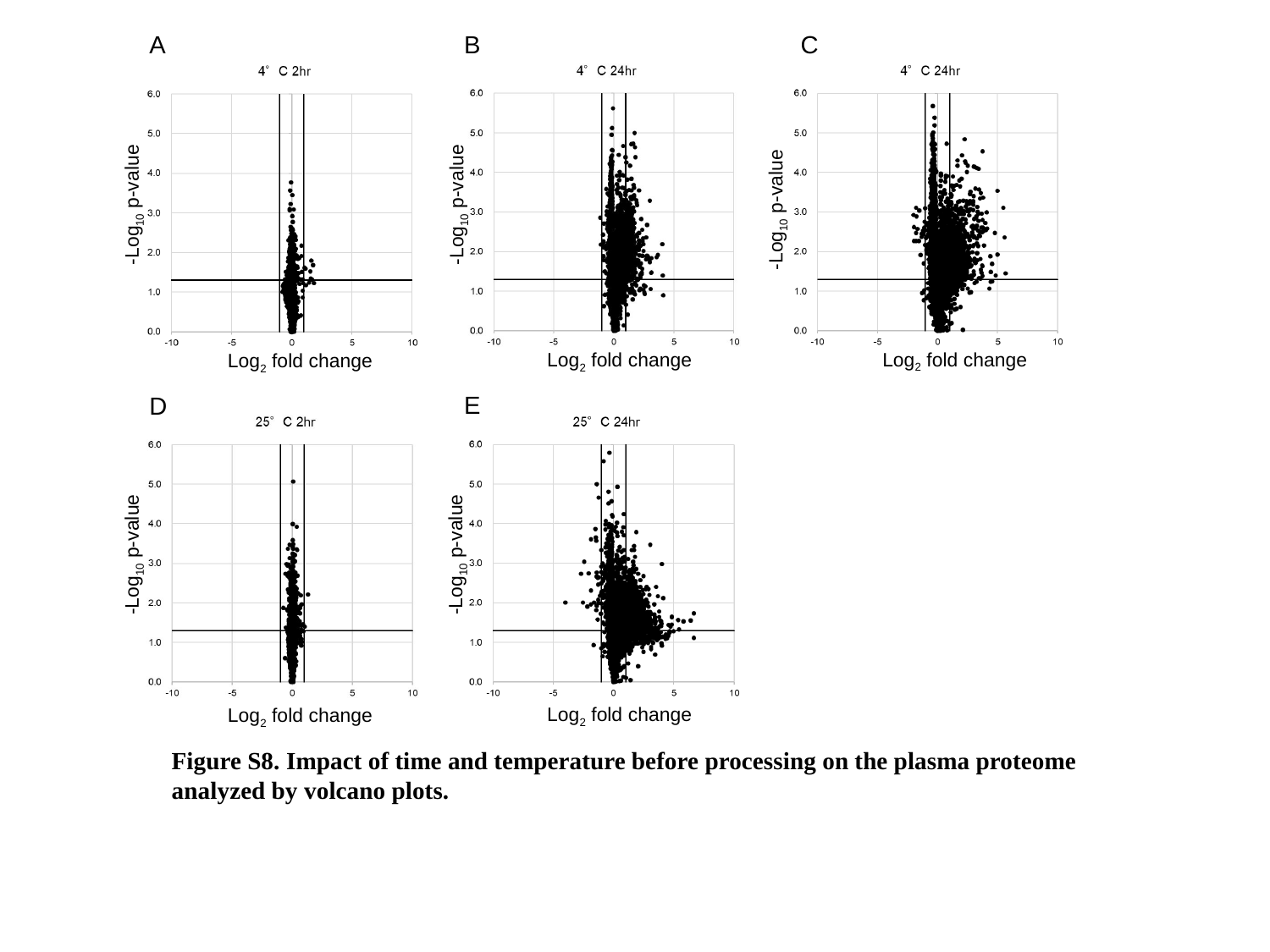

B
C
A
-Log10 p-value
-Log10 p-value
-Log10 p-value
Log2 fold change
Log2 fold change
Log2 fold change
E
D
-Log10 p-value
-Log10 p-value
Log2 fold change
Log2 fold change
Figure S8. Impact of time and temperature before processing on the plasma proteome analyzed by volcano plots.
