# Supplementary material for: The effect of preanalytical factors on cerebrospinal fluid and plasma proteomics: a systematic experimental study
Source: Clin Proteomics. 2026 May 22;23:40. doi: 10.1186/s12014-026-09604-5 (PMC13383461; doi:10.1186/s12014-026-09604-5)
Supplement: Supplementary file 10 — Supplementary Material 9: Figure S9. GO enrichment analysis of plasma proteins altered after 24 h incubation at 25°C. Plasma samples were incubated at 25°C for 24 hours prior to processing and compared with the corresponding baseline condition. Gene Ontology (GO) enrichment analysis was performed on proteins that showed large change. The dot plot displays enriched biological processes, with the x-axis representing the gene ratio (enrichment strength) and the y-axis indicating GO terms. Dot size corresponds to the number of proteins associated with each term, and color indicates the adjusted p-value (Benjamini–Hochberg correction). [file 12014_2026_9604_MOESM10_ESM.pptx]

## Slide 1
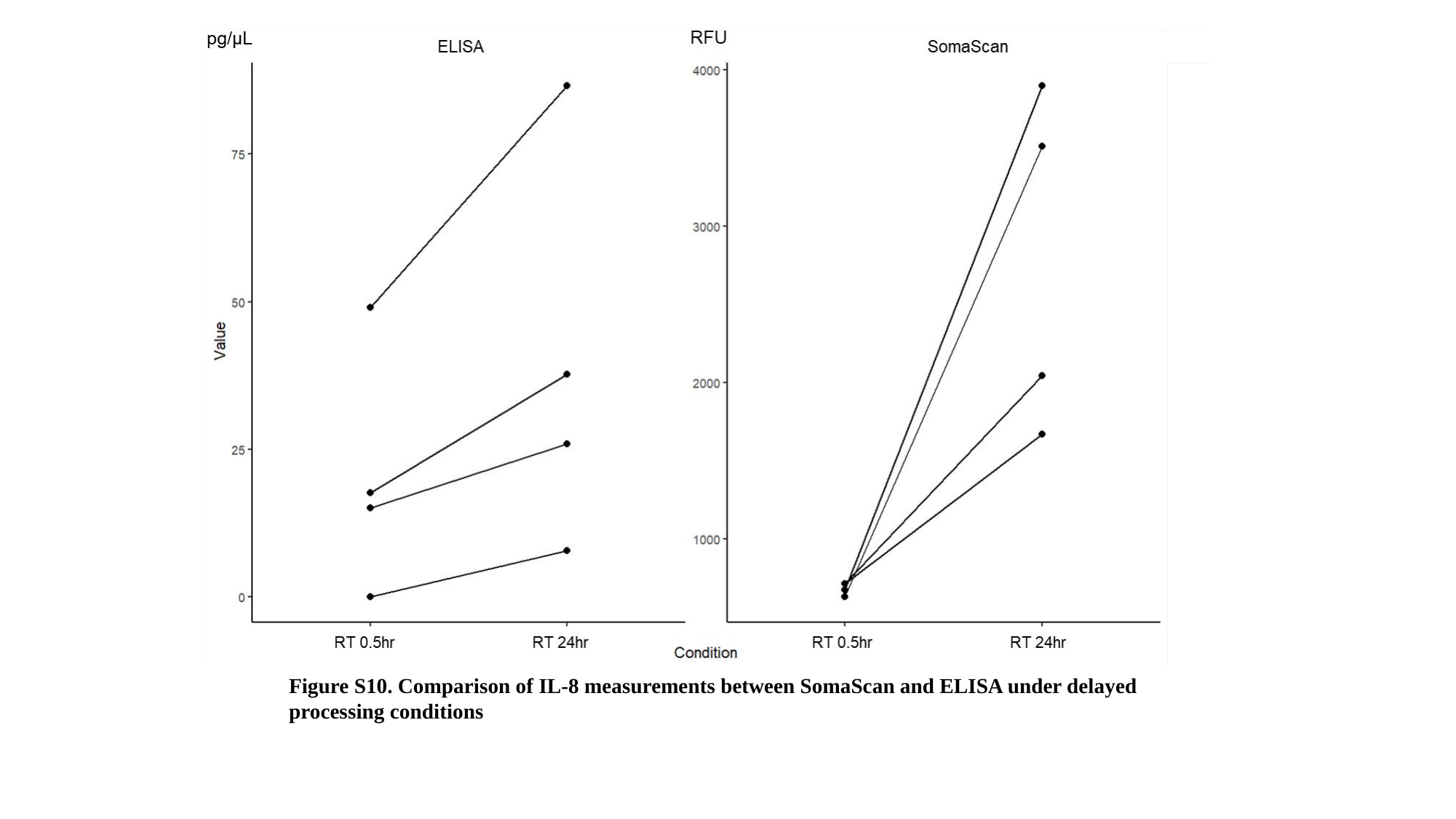

Figure S10. Comparison of IL-8 measurements between SomaScan and ELISA under delayed processing conditions
