# Supplementary material for: The effect of preanalytical factors on cerebrospinal fluid and plasma proteomics: a systematic experimental study
Source: Clin Proteomics. 2026 May 22;23:40. doi: 10.1186/s12014-026-09604-5 (PMC13383461; doi:10.1186/s12014-026-09604-5)
Supplement: Supplementary file 11 — Supplementary Material 10: Figure S10. Comparison of IL-8 measurements between SomaScan and ELISA under delayed processing conditions. Plasma samples were incubated at 25°C for 24 hours prior to centrifugation and compared with the corresponding baseline condition. IL-8 concentrations were measured using both SomaScan (right panel) and ELISA (left panel). Paired measurements from the same samples are connected by lines, illustrating changes between baseline (“RT 0.5hr”) and delayed processing (“RT 24hr”) conditions. Because one baseline ELISA measurement was below the limit of detection, fold change could not be calculated for all samples. Therefore, results are presented as paired plots rather than fold changes to allow inclusion of all data points. Despite differences in measurement scale between platforms, consistent directional increases in IL-8 were observed across samples. (A–C) CSF samples kept at 4°C for 2, 4, or 24 h before processing. (D–F) CSF samples kept at 25°C for 2, 4, or 24 h before processing. [file 12014_2026_9604_MOESM11_ESM.pptx]

## Slide 1
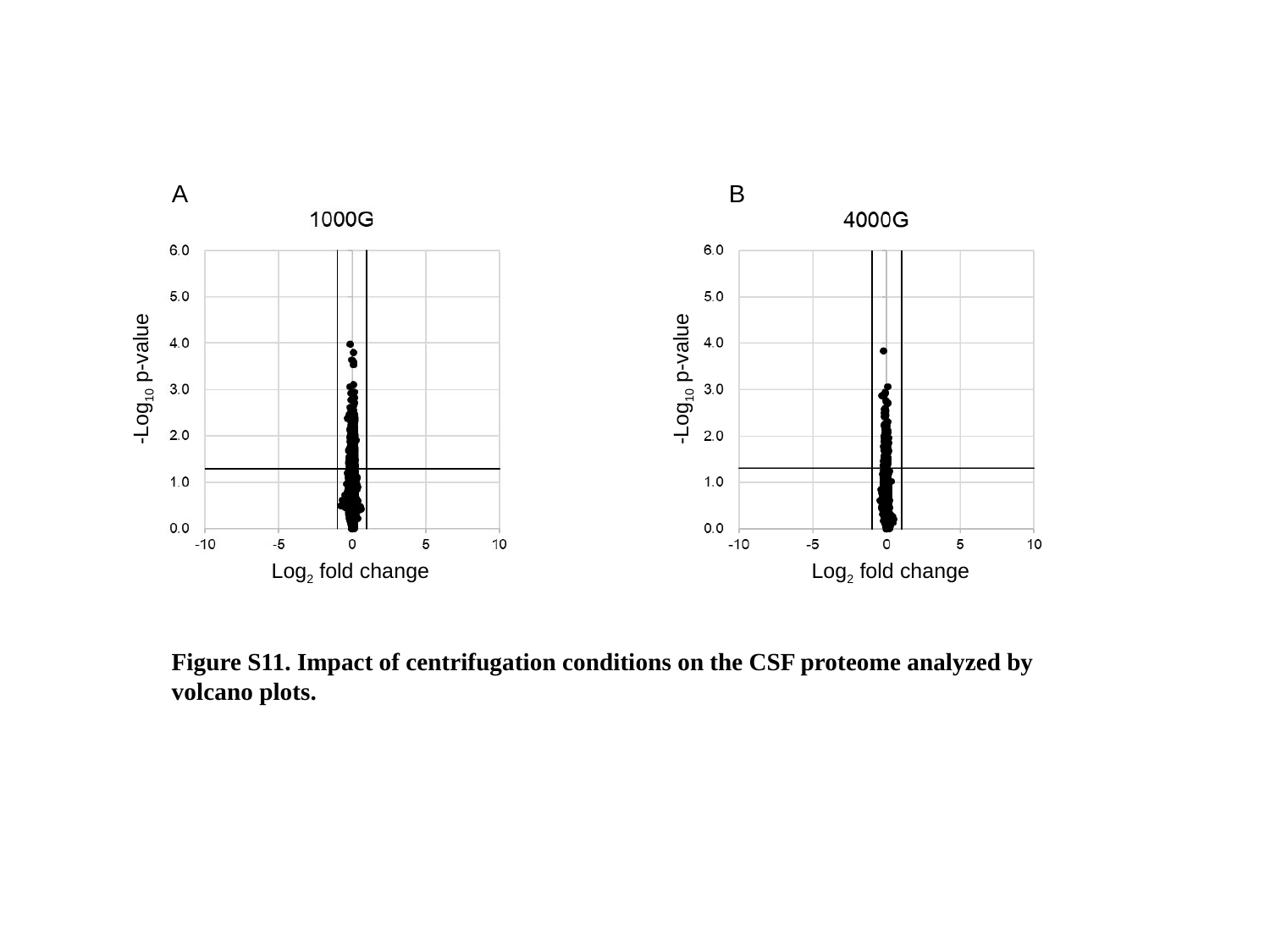

B
A
-Log10 p-value
-Log10 p-value
Log2 fold change
Log2 fold change
Figure S11. Impact of centrifugation conditions on the CSF proteome analyzed by volcano plots.
