# Supplementary material for: The effect of preanalytical factors on cerebrospinal fluid and plasma proteomics: a systematic experimental study
Source: Clin Proteomics. 2026 May 22;23:40. doi: 10.1186/s12014-026-09604-5 (PMC13383461; doi:10.1186/s12014-026-09604-5)
Supplement: Supplementary file 12 — Supplementary Material 11: Figure S11. Impact of centrifugation conditions on the CSF proteome analyzed by volcano plots. CSF samples were centrifuged at 1000 g, 2000 g, or 4000 g for 10 minutes, and the resulting supernatants were stored for analysis. Volcano plots were generated to compare each centrifugation condition with the baseline condition of 2000 g. Axes and statistical analyses are as described in Figures S2. A: Volcano plot comparing CSF samples centrifuged at 1000 g with those centrifuged at 2000 g. B: Volcano plot comparing CSF samples centrifuged at 4000 g with those centrifuged at 2000 g. [file 12014_2026_9604_MOESM12_ESM.pptx]

## Slide 1
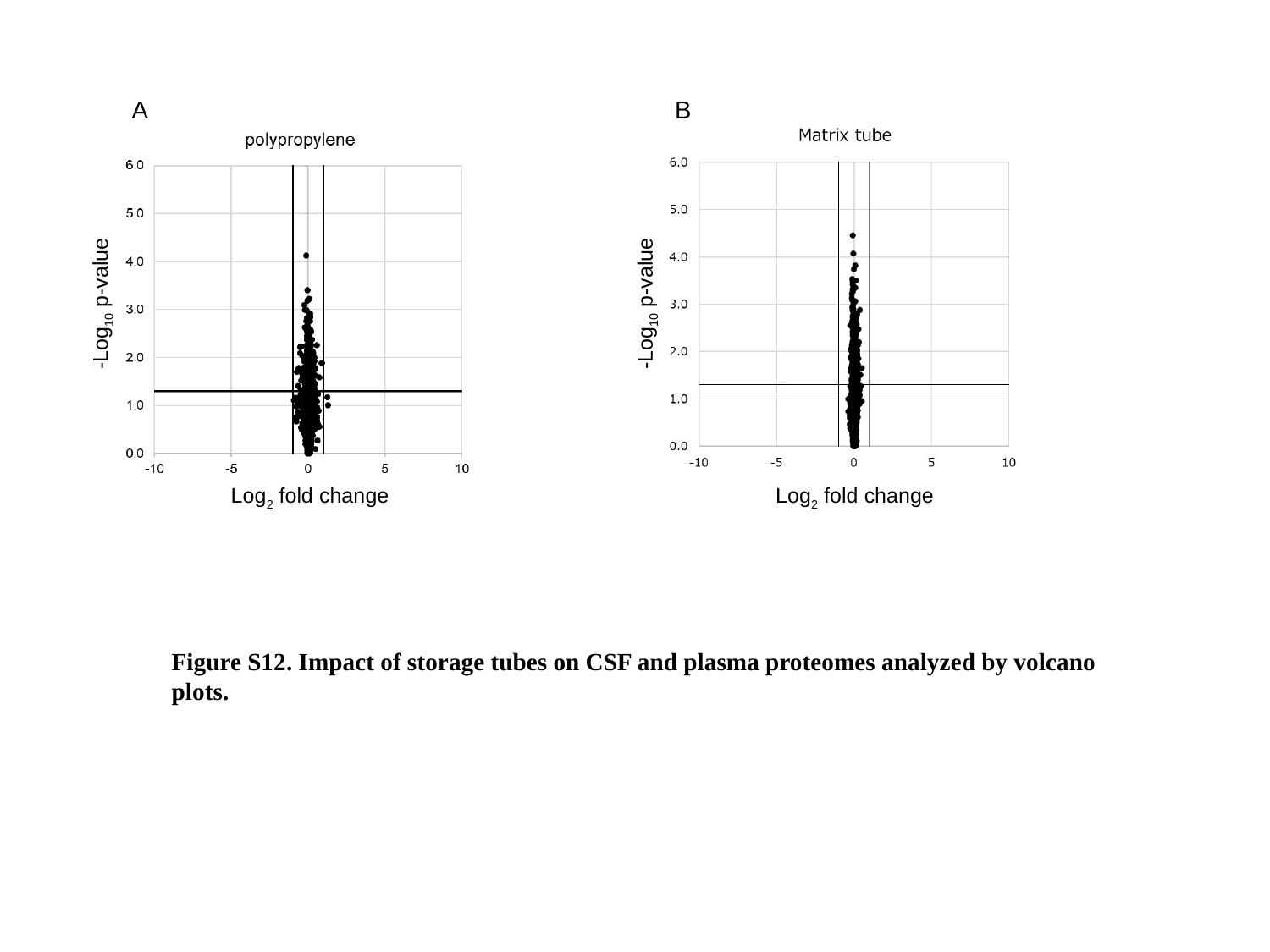

B
A
-Log10 p-value
-Log10 p-value
Log2 fold change
Log2 fold change
Figure S12. Impact of storage tubes on CSF and plasma proteomes analyzed by volcano plots.
