# Supplementary material for: The effect of preanalytical factors on cerebrospinal fluid and plasma proteomics: a systematic experimental study
Source: Clin Proteomics. 2026 May 22;23:40. doi: 10.1186/s12014-026-09604-5 (PMC13383461; doi:10.1186/s12014-026-09604-5)
Supplement: Supplementary file 13 — Supplementary Material 12: Figure S12. Impact of storage tubes on CSF and plasma proteomes analyzed by volcano plots. Volcano plots were generated to assess the impact of different storage tubes on cerebrospinal fluid (CSF) and plasma proteomes. Axes and statistical analyses are as described in Figures S2. A: CSF samples processed under identical conditions were aliquoted into polypropylene tubes (96 Jacket tubes, FCR & Bio) or low protein-binding tubes (PROTEOSAVE SS 1.5 mL Slimtube, Sumitomo Bakelite). The volcano plot compares CSF samples aliquoted into polypropylene tubes with those aliquoted into low protein-binding tubes (baseline condition). B: After centrifugation, plasma samples were aliquoted into 96 Jacket tubes (FCR & Bio) or Matrix tubes (Thermo Fisher Japan). The volcano plot compares plasma samples aliquoted into Matrix tubes with those aliquoted into 96 Jacket tubes (baseline condition). [file 12014_2026_9604_MOESM13_ESM.pptx]

## Slide 1
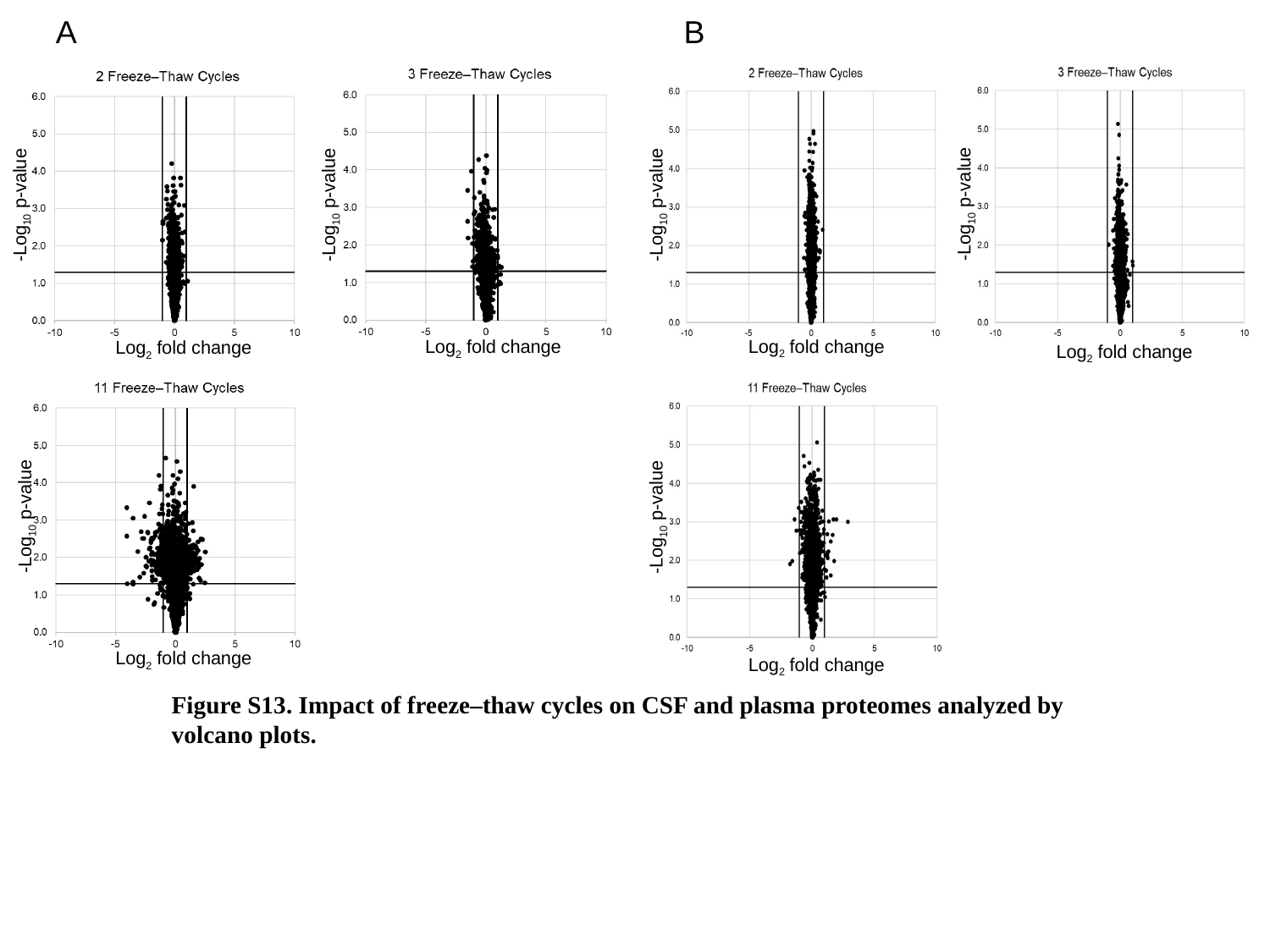

A
B
-Log10 p-value
-Log10 p-value
-Log10 p-value
-Log10 p-value
Log2 fold change
Log2 fold change
Log2 fold change
Log2 fold change
-Log10 p-value
-Log10 p-value
Log2 fold change
Log2 fold change
Figure S13. Impact of freeze–thaw cycles on CSF and plasma proteomes analyzed by volcano plots.
